# Molecular basis for transposase activation by a dedicated AAA+ ATPase

**DOI:** 10.1038/s41586-024-07550-6

**Published:** 2024-06-26

**Authors:** Álvaro de la Gándara, Mercedes Spínola-Amilibia, Lidia Araújo-Bazán, Rafael Núñez-Ramírez, James M. Berger, Ernesto Arias-Palomo

**Affiliations:** 1 Centro de Investigaciones Biológicas Margarita Salas, CSIC, Madrid, Spain; 2 Department of Biophysics and Biophysical Chemistry, Johns Hopkins University School of Medicine, Baltimore, MD USA

**Keywords:** Cryoelectron microscopy, Transposition

## Abstract

Transposases drive chromosomal rearrangements and the dissemination of drug-resistance genes and toxins^1–3^. Although some transposases act alone, many rely on dedicated AAA+ ATPase subunits that regulate site selectivity and catalytic function through poorly understood mechanisms. Using IS*21* as a model transposase system, we show how an ATPase regulator uses nucleotide-controlled assembly and DNA deformation to enable structure-based site selectivity, transposase recruitment, and activation and integration. Solution and cryogenic electron microscopy studies show that the IstB ATPase self-assembles into an autoinhibited pentamer of dimers that tightly curves target DNA into a half-coil. Two of these decamers dimerize, which stabilizes the target nucleic acid into a kinked S-shaped configuration that engages the IstA transposase at the interface between the two IstB oligomers to form an approximately 1 MDa transpososome complex. Specific interactions stimulate regulator ATPase activity and trigger a large conformational change on the transposase that positions the catalytic site to perform DNA strand transfer. These studies help explain how AAA+ ATPase regulators—which are used by classical transposition systems such as Tn7, Mu and CRISPR-associated elements—can remodel their substrate DNA and cognate transposases to promote function.

## Abstract (web summary)

Solution and cryogenic electron microscopy studies using IS*21* as a model transposase system show how AAA+ ATPases induce structural changes to prime target DNA and activate their associated transposases.

## Main

Transposable elements are DNA segments capable of moving between different locations^1^. They are widespread across all domains of life and have significantly affected genome diversity and evolution^2^. Transposable elements can modulate gene expression, disseminate virulence and antibiotic resistance cassettes and are associated with numerous diseases^3,4^. Some mobile elements are also emerging as potential biotechnological and gene editing tools^5,6^.

How transposases, which frequently assemble into modular complexes termed transpososomes to perform strand-transfer reactions, are regulated to prevent deleterious DNA breaks and recombination events has been a long-standing question^7–9^. Several transposases rely on transposon-encoded regulators, including molecular matchmakers such as those found in Tn7, bacteriophage Mu and CRISPR RNA-guided elements^7^, which belong to the ATPases associated with various cellular activities (AAA+) family of ATPases^8^. These proteins typically feature a bipartite ATPase site that is pivotal for their multimeric assembly^8^. For instance, in bacteriophage Mu, the MuB ATPase forms filaments crucial for MuA transposase activity^9^. Meanwhile, the TnsC ATPase in classical and CRISPR-associated Tn7 elements self-assembles into filaments, rings or split-rings to recruit TnsB transposase^10–14^. In addition to supporting transposase function, many regulatory factors seem to induce target immunity, a process that redirects the transposase away from its transposon sequence to prevent self-destruction^15,16^. Despite these general insights, the mechanistic principles by which transposon-encoded ATPases operate as molecular switches to both prime target DNAs and activate their associated transposases have remained unclear.

**Fig. 1:**
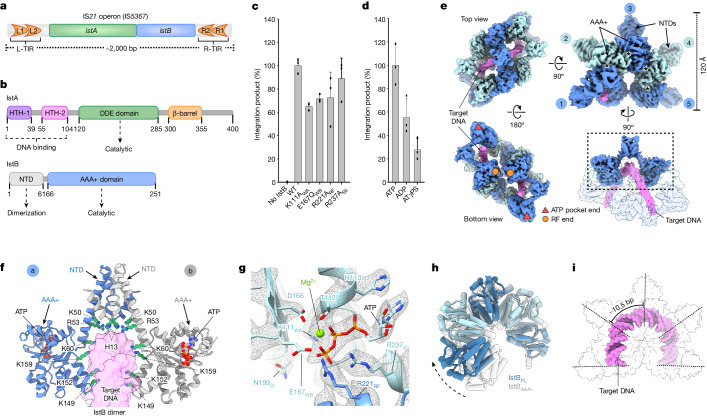
IS*21* organization and structural and functional characterization of IstB. **a**, IS*21* genetic organization (including the IS*5367* family member). L-TIR and R-TIR denote left and right TIRs, respectively. **b**, Domain organization of IstA and IstB. NTD, N-terminal domain. **c**, In vitro DNA integration activity of IstB WT and ATPase mutants. Integration represented by the amount of linear DNA product appearing over 30 min. SII, sensor II; RF, arginine finger; WA, Walker A; WB, Walker B. Data are mean ± s.d. (*n* = 3 independent replicates). Dots indicate individual values. **d**, Effect of different nucleotides in the integration reaction. Mean ± s.d. (*n* = 3 independent replicates). Dots indicate individual values. **e**, Unsharpened cryo-EM maps (threshold 0.0147) of the complex of IstB–ATP–target DNA. The oligomer is composed of five dimers (numbered blue circles, shaded in two blue tones) and sequesters curved target DNA (violet). Asterisks mark nucleotide-binding pockets. Bottom-left view shows free RF ends (orange circles) and the incomplete ATP pocket ends (red triangles). **f**, IstB dimer. Each monomer is coloured differently. Positively charged resides that interact with the target DNA are shown as green sticks. **g**, Close-up view of a representative nucleotide-binding pocket located between two neighbouring monomers (coloured in different blue tones). A sharpened map is shown as a mesh (threshold 0.037). **h**, The helical pitch and curvature of full-length IstB is more expanded than that observed for the isolated AAA+ domain (Protein Data Bank (PDB) identifier 5BQ5)^27^. **i**, Spacing between IstB dimers roughly follows the periodicity of target DNA, introducing an approximate 180° bend into the duplex. Source Data

The smallest and most abundant type of DNA transposons only contains proteins implicated in the transposition reaction. In bacteria, these are termed insertion sequences (ISs)^17^. The IS*21* family is particularly widespread, has been found in clinically important multidrug-resistant strains and has shaped the evolution of human pathogens^18,19^. Owing to their simplicity and robust activity, IS*21* elements are a particularly appealing model for mechanistic studies^20,21^. The archetypal IS*21* sequence contains two terminal inverted repeats (TIRs) that are typically composed of at least two repeated elements (L1 and L2 for the left ends, and R1 and R2 for the right ends)^20^ (Fig. 1a). Additionally, IS*21* encodes two essential proteins, IstA and IstB, which are required for transposition^22^ (Fig. 1b).

The IstA transposase possess two helix-turn-helix (HTH) DNA-binding domains, a DDE catalytic motif and a β-barrel followed by a flexible carboxy-terminal region^21^ (Fig. 1b). It has been reported that IstA, the domain organization of which is similar to that of the MuA and TnsB transposases, can oligomerize into a highly intertwined tetramer that synapses two transposon ends into a supercoiled configuration^21^. However, this tetramer state is inactive and requires the regulatory IstB factor to perform transposition^21,23^. IstB, similar to the MuB and TnsC helper proteins, is a member of the AAA+ family and a close paralogue of the DnaC/I helicase loader. It is composed of a small amino-terminal extension (different from those found in TnsC and MuB) followed by an ATPase domain^24–27^ (Fig. 1b). Although previous low-resolution studies have shown that IstB self-assembles into a pentamer-of-dimers oligomer that clamps around and bends a target DNA duplex^27^, how the protein dimerizes through its N-terminal domain and specifically engages DNA to enforce curvature has not been established. How IstB recruits the IstA transposase to DNA to stimulate strand-transfer activity is similarly not known.

To address these and other questions involving IstAB function, we combined biochemical and structural methods to define in molecular detail the nucleotide-dependent transposition reaction of IS*21*. In vitro integration assays highlight the relevance of the nucleotide and ATP-binding motifs of IstB in the transposition process. A 3.2 Å resolution structure of IstB bound to target DNA reveals how specific elements of the N-terminal dimerization and AAA+ ATPase domains facilitate protein oligomerization to engage and bend a 60 bp duplex while maintaining an ATPase-repressed state. An accompanying 3.6 Å resolution structure of a complete strand-transfer complex shows that two IstB decamers can homodimerize to stabilize the target DNA into a kinked S-shaped platform that engages an IstA transposase tetramer that forms a nearly 1 MDa synaptic particle. The C-terminal domains of IstA interact with a pair of flanking IstB subunits to favour the adoption of an ADP state. Meanwhile, reorganization of the IstA tetramer into an extended conformation that positions the donor DNA molecule and IstA active sites in a catalytically competent configuration to perform strand-transfer and DNA integration is also triggered. Our findings, together with comparative studies, help explain how DNA transposition can be controlled by nucleotide-dependent matchmaker proteins and highlight how a common AAA+ ATPase scaffold can be repurposed by different transposase and DNA replication systems to differentially engage and remodel client protein and duplex DNA substrates in a functionally distinct manner.

## IstB AAA+ promotes IS*21* transposition

**Extended Data Fig. 1:**
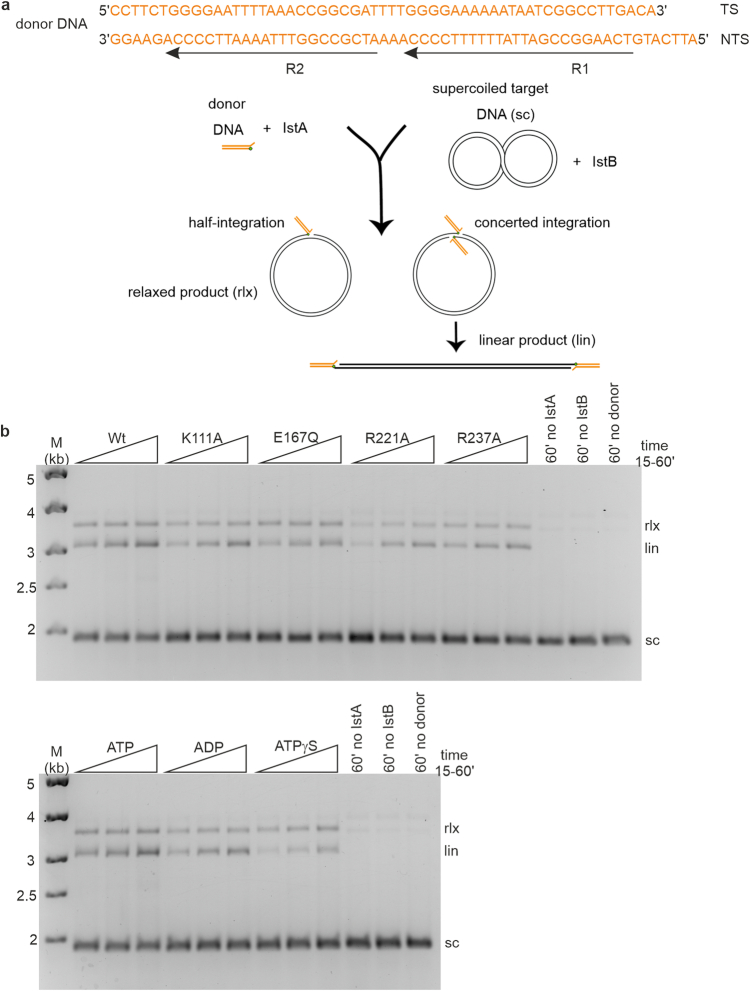
Schematic of an in vitro integration assay and effects of IstB ATPase mutants and nucleotides on the integration reaction. **a**, Pre-cleaved donor DNA includes the sequence of the transposon end (orange; TS, transferred strand; NTS, non-transferred strand). The donor duplex, containing a terminal CA dinucleotide with a reactive 3’-OH (green), can be integrated by IstA into a supercoiled target plasmid (black) in the presence of IstB. The reaction produces relaxed and linearized plasmids as result of half or concerted integration, respectively. **b**, Representative native agarose gel of the effect of IstB mutants and nucleotides on the integration reaction (*n* = 3 independent experiments). For gel source data, see Supplementary Fig. 1.

Previous studies have established that the IstB ATPase, similar to TnsC and MuB^25,26,28^, is essential for stimulating DNA transposition by its cognate transposase^21,23^. Although ATP is required to facilitate proper IstB assembly^27^, the precise role of nucleotide binding and hydrolysis in IstA recruitment, activation and DNA transposition has been unclear. We therefore sought to better resolve how IstB catalytic activity controls strand transfer by IstA. First, we purified a panel of ATPase mutants known to be crucial for AAA+ activity (for example, Walker A, Walker B, arginine finger (RF) and sensor II)^8^ from a prototypical IS*21* family member, IS*5376*, found in *Geobacillus stearothermophilus*^20,27,29^ and tested them in an integration assay. In brief, the transposase was incubated with 55 bp donor DNA duplexes containing the complete sequence of the right TIR (Extended Data Fig. 1a). Note that it has previously been shown that IstA exhibits similar levels of in vitro activity using either the isolated right TIR or an equimolar mixture of left and right TIRs^21^. The donor molecule comprised both a transferred strand, pre-cleaved at a characteristic CA dinucleotide that is typically attacked by transposases and retroviral integrases, and a non-transferred strand bearing a short (5 nucleotide (nt)) 5′ overhang. Similar to MuA^30^, the presence of a flanking 5′ overhang stimulates integration by IstA^21^. The transposase–donor mix was then combined with a supercoiled target plasmid in the presence of nucleotide and the different IstB variants. The integration of one donor duplex generated a relaxed plasmid. By contrast, the concerted integration of two TIRs, the product of a complete DNA transposition reaction, resulted in a linearized plasmid (Extended Data Fig. 1).

In agreement with previous studies^21,23^, the integration assays showed that IstB is necessary to perform strand transfer (Fig. 1c, Extended Data Fig. 1b and Supplementary Fig. 1). Notably, ATPase-deficient mutants still supported activity but to differing extents (Fig. 1c, Extended Data Fig. 1b and Supplementary Figs. 1 and 2), a finding consistent with results reported for analogous mutants of MuB and TnsC^31–33^. We next sought to further define the role of the nucleotide in the transposition process. We repeated our transposition experiments with wild-type IstB in the presence of both ADP and the slowly hydrolysable ATP analogue ATPγS (the latter of which supports IstA interactions^27^). Under these conditions, the integration reaction still proceeded but was again compromised, particularly by ATPγS (Fig. 1d and Extended Data Fig. 1b). Overall, these results demonstrate that the nucleotide turnover cycle has an important (albeit not indispensable) role in allowing IstB to support transposition by IstA.

## Autoinhibited IstB remodels duplex DNA

**Extended Data Fig. 2:**
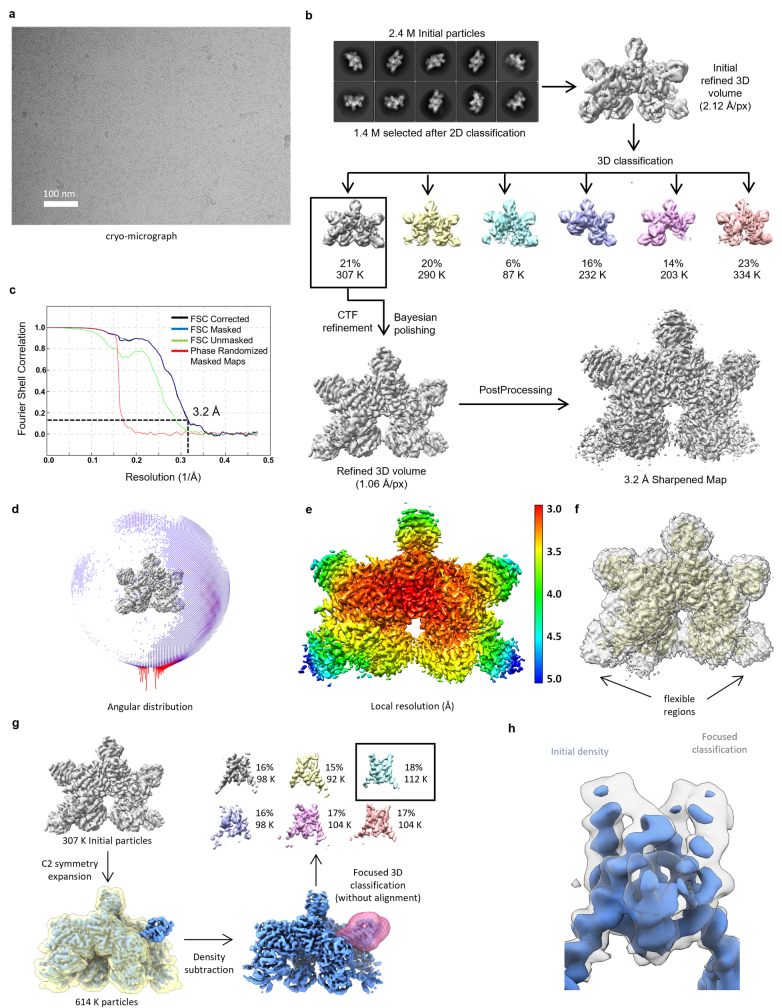
IstB workflow and image processing. **a**, Representative cryo-EM micrograph of the IstB•ATP•target-DNA complex. Scale bar (white, bottom left) represents 100 nm. The micrograph shown is an example image from a full dataset consisting of 6,214 micrographs. **b**, Image processing workflow. **c**, Fourier Shell Correlation (FSC) of the final cryo-EM map. **d**, Angular distribution indicating the range of observed particle orientations. **e**, Local resolution of the sharpened EM density. **f**, Comparison of the unsharpened map at 2 different thresholds (0.0457 and 0.0207 in gold and grey, respectively), showing the flexible nature of the IstB terminal dimers. **g**, Processing workflow showing the masks used for density subtraction (yellow) and focused 3D classification (violet). **h**, Comparison of the N-terminal domain before (blue) and after (transparent density) symmetry expansion and focused 3D classification. Both maps displayed at a threshold of 0.0226.

It has been noted that the N-terminal domain and the AAA+ module of IstB are both required for nucleotide-controlled self-assembly^27^. However, the limited resolution (about 9 Å) of reconstructions available so far have precluded an understanding of how IstB specifically engages DNA and how its active sites are prevented from hydrolysing ATP in an oligomerized state. To resolve these questions, cryogenic electron microscopy (cryo-EM) was used to determine a structure of IstB bound to DNA in the presence of ATP. A 3.2 Å resolution structure was obtained, and the quality of the cryo-EM map readily permitted rebuilding of a docked crystal structure of the AAA+ ATPase region of IstB^27^. We also performed de novo modelling of the N-terminal region of the protein and the target nucleic acid, which generated a complete model of the complex (Fig. 1e, Extended Data Fig. 2 and Supplementary Video 1).

**Extended Data Fig. 3:**
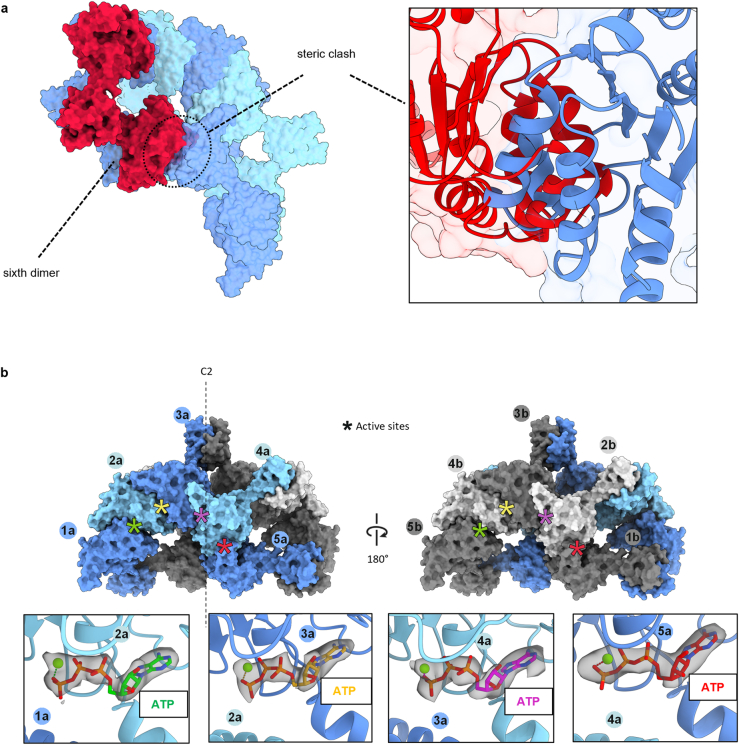
IstB ATPase oligomerization is limited to a pentamer of dimers with 8 competent sites occupied by ATP. **a**, IstB assembly is limited to five dimers. The modeling of a sixth dimer (red) to the decameric complex, based on propagating the AAA + /AAA+ interactions seen between other IstB protomers, generates a steric clash that, at least in these conditions, prevents IstB from forming continuous filaments. **b**, IstB active site is composed by catalytic residues located in two adjacent subunits. Four catalytic competent ATPase pockets (asterisks) are formed by monomers 1a-5a (blue), whereas monomers 1b-5b (grey) form other four symmetry-related ATPase sites (target DNA omitted for clarity). Bottom images: Close-up views of complete nucleotide binding pockets of IstB reveal that all are occupied by ATP (only 1a-5a sites are shown since the symmetry-related formed by monomers 1b-5b are identical).

In accord with previous low-resolution studies^27^, the cryo-EM structure showed that IstB utilizes its N-terminal domains to form dimers that oligomerize into a clamshell-shaped, two-fold symmetric decamer through ATP-dependent AAA+ domain interactions (Fig. 1e,f). This result was in sharp contrast to the polar rings and filaments observed for other transposition-related ATPases. Although IstB octamers (tetramers of dimers) were observed during image processing, the cryo-EM reconstruction confirmed why subunit assembly is limited to five IstB dimers. That is, the inverted arrangement of two AAA+ domain pentamers, the DNA-binding surfaces of which face one another owing to the molecular two-fold axes of their associated N-terminal domains (Fig. 1e), sterically prevents the binding of a sixth ATPase subunit to the complex in this configuration (Extended Data Fig. 3a). Thus, the N-terminal region is not only responsible for dimerizing IstB protomers but also for preventing the formation of contiguous ATPase oligomers as seen with TnsC and MuB.

In the IstB decamer, eight out of the ten nucleotide-binding sites adopt a canonical AAA+ ATPase configuration^8^. This is characterized by the interaction of a positively charged residue of one monomer—the RF—with the Walker A and Walker B signature motifs from a neighbouring subunit (Fig. 1e,g). Close inspection of the EM map showed clear density maps for ATP in all binding pockets (Fig. 1g and Extended Data Fig. 3b). Exceptions were the two terminal monomers where the nucleotide-binding sites are solvent exposed (the outer dimers are also more flexible; Extended Data Fig. 2f). Notably, although the catalytic residues of the eight complete active sites seemed to contain all the key residues for nucleotide hydrolysis (Fig. 1g), full-length IstB has limited ATPase activity on its own^27^. The previous IstB structure could not account for this observation because of the low level of detail in the reconstruction. However, the higher resolution maps resolved here established that the pitch and curvature that the AAA+ domains adopt in the decamer are different from that seen in a crystal structure of the isolated ATPase fold, which was obtained in the presence of a non-hydrolysable analogue (Fig. 1h). Owing to the absence of regulatory domains and to the similarity with classical AAA+ members, the X-ray derived model probably represents an ideally organized active site^27^. It has been noted that for related factors, such as the polymerase clamp loaders, both large-scale and subtle orientation changes between AAA+ subunits can alter the interaction of the RF with the nucleotide, which significantly affects ATPase activity^34^. Consistent with this observation, the change in rotation and tilt of the IstB monomers led to a misalignment of key catalytic residues, which maintains the protein in an autoinhibited configuration.

The ATPase domains of IstB are stabilized by the nucleotide-dependent AAA+ interactions that mediate the formation of the pentameric complex. By contrast, the N-terminal regions of the protein showed a higher degree of flexibility owing to their external position and reduced number of contacts with the rest of the particle (Extended Data Fig. 2e,f). Nonetheless, we were able to improve the density of this domain using symmetry expansion and 3D classification, which permitted the building of an atomic model (Fig. 1f and Extended Data Fig. 2g,h). The first 65 residues of IstB are composed of 3 small α-helices, which form the dimerization interface of the protein and connect through an elongated helical extension to the AAA+ domain (Fig. 1f). Close inspection of the complex revealed the presence of numerous positively charged residues that interact with the target DNA (Fig. 1f and Supplementary Video 1), which partially explained the 30-fold increase in duplex affinity observed for the full-length protein compared with the isolated ATPase module (*K*_d,app_ of 40 nM versus 1.5 µM, respectively)^27^. These amino acids, together with a conserved region of the AAA+ domains known as the initiator-specific motif, form an internal cavity within each IstB dimer that engages DNA primarily through nonspecific contacts to the phosphodiester backbone. The subsequent assembly of IstB dimers into the final decameric state in turn generates a continuous, U-shaped channel that tracks the periodicity of the duplex DNA (about 10.5 bp) and introduces an approximate 180° curve into the target molecule (Fig. 1i). Taken together, these results show that IstB utilizes its N-terminal extension to not only facilitate protein oligomerization and target duplex bending but also maintain the AAA+ domains in an inactive configuration that stabilizes the assembly on DNA while preventing futile ATP hydrolysis cycles.

## IS*21* transpososome reconstitution

**Fig. 2:**
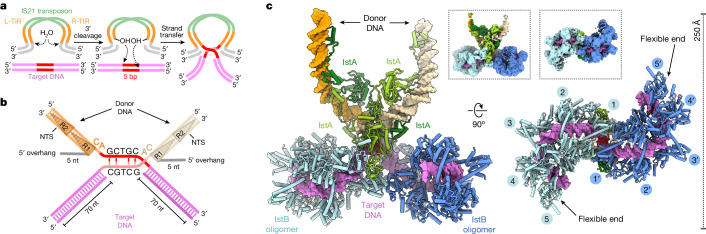
IstA–IstB STC organization. **a**, Schematic of the IS*21* transposition mechanism. **b**, Simplified diagram of the STC DNA used for cryo-EM. The nucleic acid is designed to mimic the product of the strand-transfer reaction. The catalytic CA dinucleotides and the positions of the terminal inverted repeats (R1 and R2) of the donor DNA are indicated. NTS, non-transferred strand. **c**, Two orthogonal views of the IstA–IstB STC (holo-transpososome) structure. The five dimers in each IstB oligomer are identified with numbered circles (each IstB decamer in a different blue shade). Images in the boxes show unsharpened maps of the corresponding views (0.00228 threshold).

**Extended Data Fig. 4:**
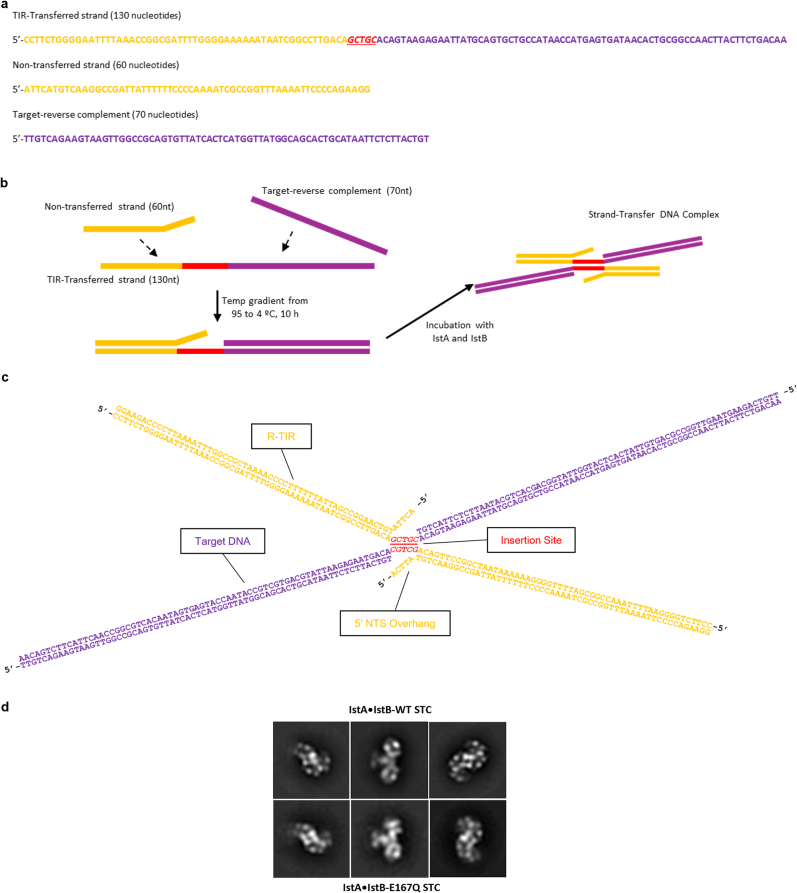
Strand Transfer Complex design and assembly. **a**, The DNA substrate is composed of three different single-stranded DNA sequences. The longest molecule (130 nt) contains the complete sequence of the right TIR (orange) covalently bound to the target DNA (violet), including the insertion sequence (red). The second strand (60 nt) contains the complementary sequence of the right TIR (non-transferred strand, NTS), including a 5 nt-long 5’ overhang. The third strand (70 nt) contains the nucleotide sequence complementary to the target DNA. **b**, Step-wise formation of the substrate. Half-substrates are formed mixing and gently annealing the three DNA strands in equimolar amounts. The annealed substrate dimerizes via the insertion site aided by the proteins. **c**, Schematic of the final substrate displaying the complete sequence. **d**, Negative staining reference-free 2D averages show that the STC complex formed with wild-type IstB and the E167Q mutant are virtually identical at this resolution.

To better understand how IstB supports the recruitment and activation of IstA, we reconstituted both proteins bound to a strand-transfer DNA, an approach that has been widely used for other transposases and retroviral integrases^35–38^. IS*21* elements are thought to follow a general catalytic pathway similar to that described for most transposition systems^17,20,39^. In this scheme, the IstA transposase first pairs and cleaves the transposon ends to generate two free 3′ OH groups (Fig. 2a). The enzyme then catalyses a nucleophilic attack (generating two staggered cuts) to integrate the donor molecules into the target duplex, which results in a DNA product referred to as a strand-transfer complex (STC). In vitro, IstA exhibits similar levels of activity and 3D structure when incubated with only the isolated right transposon end compared with an equimolar mixture of left and right ends^21^. Hence, to reduce structural asymmetry, a 130-bp-long donor DNA was generated that contains two copies of the right donor transposon sequence at each end. This substrate thus includes the R1 and R2 repeats and a short 5′ overhang representing a segment of the flanking non-transferred strand, which would remain uncleaved if IS*21* uses a copy-out/paste-in replicative pathway as has been proposed^20,39^. This type of substrate design has been used in the analysis of related transposases^36,37^ (Fig. 2b and Extended Data Fig. 4). For the target molecule, because IS*21* does not have a strong target sequence specificity, we used a preferred insertion sequence that has been previously described^21^. Because numerous IS*21* members (including IS*5376*) generate 5 bp direct repeats following transposition^20,21^, the insertion sites in the target were spaced 5 bp apart (Fig. 2b and Supplementary Table 1).

**Extended Data Fig. 5:**
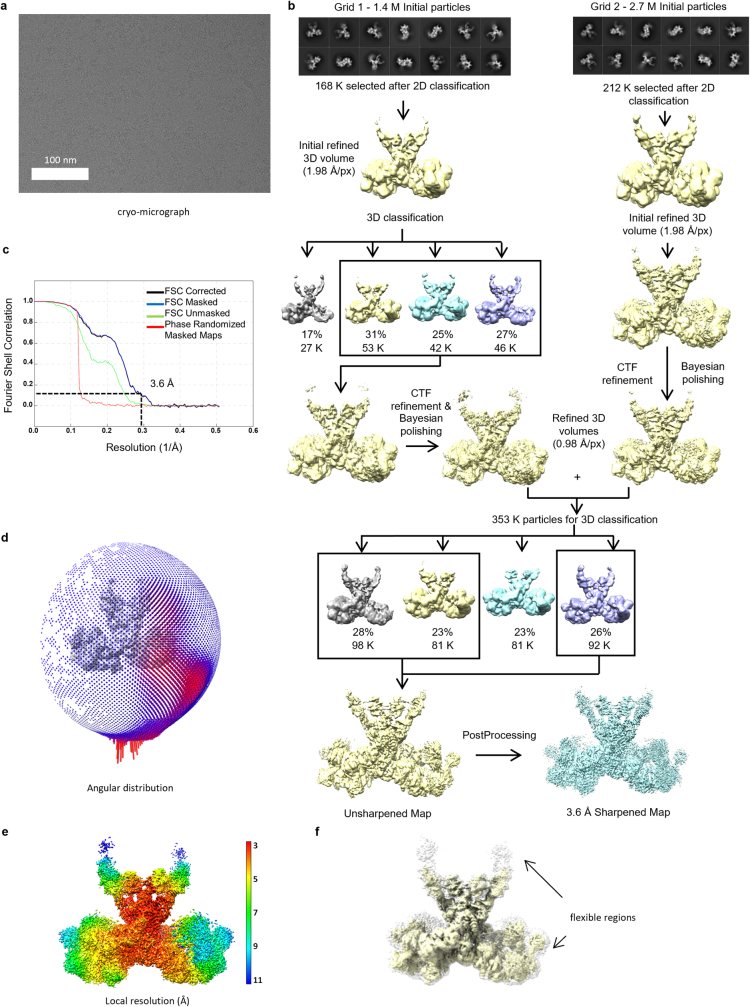
Cryo-EM imaging and image processing pipeline of the holo-transpososome complex. **a**, Representative cryo-EM micrograph from the reconstituted holo-transpososome sample. Scale bar (white, bottom left) represents 100 nm. The micrograph shown is an example image from a combined dataset consisting of 36,276 micrographs. **b**, Image processing workflow used to analyze the cryo-EM data. **c**, Fourier Shell Correlation (FSC) of the cryo-EM density map. **d**, Angular distribution plot. **e**, Local resolution of the sharpened map. **f**, Comparison of the unsharpened map at 2 different thresholds (0.00327 and 0.00144 in gold and grey, respectively) highlights the flexibility present at the distal sites of the transposase and IstB.

**Extended Data Fig. 6:**
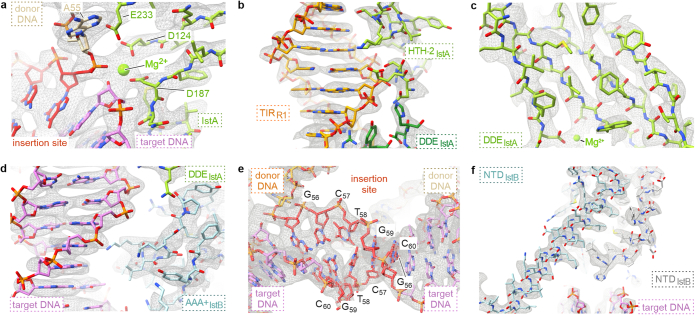
Detailed views of relevant regions of the holo-transpososome map and atomic model. **a**, IstA area around the DDE motif (sharpened density map is contoured at a threshold value of 0.00865 in all panels). **b**, Details of some contacts between the transposase and the R1 repeat from the donor DNA (TIR_R1_). **c**, Close-up view of one of the catalytic DDE domains. **d**, Contacts between IstB AAA+ domain and the target DNA near IstA catalytic monomer. **e**, Density showing the goodness of the fit of the DNA around the insertion site. **f**, Detailed view of the NTDs of a central IstB dimer.

Following the design of the DNA substrates, we used gel filtration chromatography and negative-staining EM to optimize buffer conditions, protein concentrations and protein-to-DNA ratios to maximize transpososome formation (see Methods for details). The 2D class averages showed that after incubation with substrate DNA in the presence of ATP, IstA and IstB assemble into a well-defined, high molecular weight oligomer that adopts different orientations on the grid (Extended Data Fig. 4d). Initial analysis of the sample assembled with wild-type proteins revealed that the complex was unstable, probably owing to an ability of IstA to stimulate ATP hydrolysis by IstB and promote disassembly^27^. We therefore used an IstB mutant with reduced ATPase and integration activity (Walker B; E167Q) to form a more stable complex that seemed identical to that formed by the wild-type proteins (Extended Data Fig. 4d). Although some of the distal regions of the IstAB complex showed a degree of flexibility, we were able to obtain a 3.6 Å resolution cryo-EM map after performing several rounds of 2D and 3D classification (Fig. 2c and Extended Data Fig. 5). The reconstruction of the roughly 1 MDa complex showed good quality for IstA, IstB and DNA, which enabled us to build an atomic model of the holo-transpososome (Fig. 2c and Extended Data Fig. 6).

## IstB traps an S-shaped target DNA

The cryo-EM structure showed that the IS*21* holo-transpososome is formed by one IstA tetramer and two IstB decamers (Fig. 2c and Supplementary Video 2). Although the ATPase oligomers exhibited some degree of compositional heterogeneity, due in part to the loose attachment of the most exterior IstB dimers (labelled as 4, 4′, 5 and 5′ in Fig. 2c), two pentamers of dimers could be readily modelled into the density map. Overall, the two IstB decamers formed by the Walker B mutant in the complex were highly similar to that seen for the wild-type protein on its own (1.56 Å root mean square deviation between 2,346 aligned Cα positions). This result confirmed that the ATPase-deficient mutant maintains the same protein fold and oligomeric state as the wild-type protein (Figs. 1e and 2c and Extended Data Fig. 4d).

**Fig. 3:**
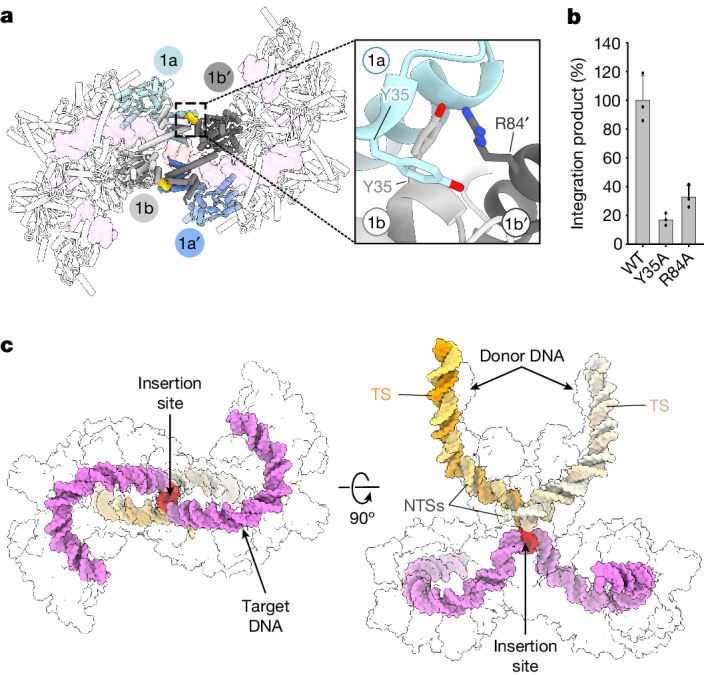
Specific interactions between the two IstB oligomers and general DNA organization in the STC. **a**, Details of the interaction between IstB oligomers in the IstA–IstB holo-transpososome. Only the monomers implicated in the two decamer–decamer contact points (gold) are shown as coloured (blue and grey) cartoons. Target DNA is shown as a transparent violet surface. The IstA-contacting dimers (1a–1b, light tone and 1a′–1b′, dark tone) are symmetrically related. Inset depicts three of the main residues that mediate the IstB–IstB interaction. **b**, Alanine mutants of residues implicated in IstB decamer dimerization display a significant loss in integration activity. Mean ± s.d. (*n* = 3 independent replicates). Dots indicate individual values. **c**, 3D organization of STC DNA. IstB remodels the target DNA (violet) into a kinked S-shaped configuration. The insertion site (5-bp long) in the target molecule is covalently attached to the donor DNA and is shown in red. Dark and light tones in the donor molecules indicate the transferred strand (TS) and NTSs, respectively. Proteins are shown as transparent surfaces for clarity. Source Data

**Extended Data Fig. 7:**
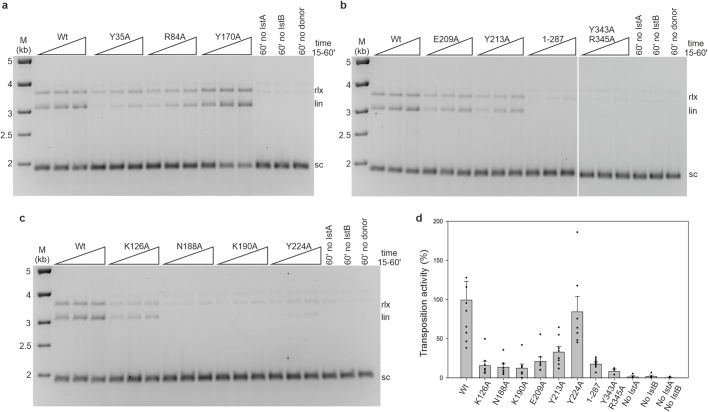
Effects of IstA and IstB mutants on the integration reaction. **a**, Representative native agarose gel of the effect of IstB mutants on the integration reaction (*n* = 3 independent experiments). For gel source data, see Supplementary Fig. 1. **b**, In vitro integration experiments with the C-terminal deletion mutant and other variants of IstA involved in the interaction with IstB. A representative native agarose gel is shown (*n* = 3 independent experiments). For gel source data, see Supplementary Fig. 1. **c**, Representative native agarose gel of in vitro integration assays with mutants of IstA implicated in the association with the target DNA (*n* = 3 independent experiments). For gel source data, see Supplementary Fig. 1. **d**, Transposition activity in a cell-free assay measured by qPCR. At least three biological replicates were performed for each IstA mutant. Individual data points representing all replicates are depicted as dots. Boxes and error bars represent the mean value and standard error, respectively. Data provided as a Source Data file. Source Data

Two IstB decamers are also seen to dimerize in the presence of the transposase. Notably, they do not assemble into a continuous filament but instead interact in a head-to-head configuration (that is, through the interior terminal dimers of each decamer, labelled 1 and 1′ in Fig. 2c). The N-terminal domains of each innermost dimer of an IstB oligomer dock against an α-helix (amino acids Glu83–Thr91) located in the dyad-proximal AAA+ domains of the opposing decamer (Fig. 3a and Supplementary Video 3). Several residues seem to participate in the interaction, including Arg84, which packs against two symmetry-related tyrosines (Tyr35) that flank the two-fold symmetry axis of the first IstB dimer (Fig. 3a). Mutation of these amino acids led to a significant disruption of IstB-stimulated DNA integration by IstA (Fig. 3b and Extended Data Fig. 7a), a result that corroborated their structural importance.

The cryo-EM reconstruction of the IS*21* transpososome showed that IstB closely tracks the duplex DNA (about one DNA turn per IstB dimer) to maintain the overall configuration and nucleic acid contacts described for the IstA-free complex. However, the interaction between the two IstB decamers also induces the DNA to adopt a sharp deformation at their junction to give rise to a kinked S-shaped configuration overall (Fig. 3c). It has been suggested that DNA sequences that are prone to kinking can serve as transposition hotspots for IS*21* and other transposon families such as IS*3* (refs. ^20,40^). Accordingly, the sharp DNA bend at the IstB decamer junction serves as the point of contact between the target substrate and the donor DNA brought to the complex by the IstA transposase (Fig. 3c and Extended Data Fig. 6a).

## IstA β-barrel stimulates ATP hydrolysis

**Fig. 4:**
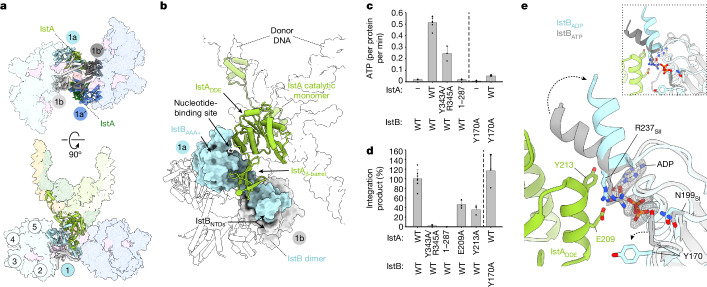
The IstA transposase C-terminal domain interaction with IstB is crucial for ATP hydrolysis and DNA integration. **a**, IstA catalytic subunits (light and dark green) interact with one monomer (1a and 1a′) from a dyad-proximal dimer of each IstB oligomer. The bottom view only shows one of these interactions for clarity. **b**, Detailed view of IstA–IstB contacts. The β-barrel of each catalytic IstA monomer (green cartoon) inserts between the NTD and AAA+ domains of one subunit of a dyad-proximal IstB dimer (1a, blue, is shown). Contacts are also formed between the DDE and AAA+ domains of IstA and IstB, respectively. DNA is shown as transparent surface. **c**, ATPase activity assays of wild type (WT) IstAB, a Y343A/R345A double mutant of IstA, a C-terminal deletion mutant of IstA (1–287), and a Y170A mutant of IstB (a dotted line separates the IstA and IstB data). Mean ± s.d. (*n* = 6 independent replicates, except for Y343A and R345A, where *n* = 3). Dots indicate individual values. **d**, In vitro integration experiments of the different IstA and IstB mutants, including those described in **c**. Mean ± s.d. (*n* = 3 independent replicates, except for WT IstAB, where *n* = 7). Dots indicate individual values. **e**, Superposition of IstA-interacting monomers of IstB (1a and 1a′), associated with ADP, and an internal IstB monomer (ATP-bound) in the holo-transpososome. Nucleotide and key residues are depicted as sticks. SI, sensor I; SII, sensor II. The density of ADP is from focused refinement of the transpososome core contoured at 0.0113. Inset shows superposition of same ADP-bound monomer with ATP-bound monomer in IstB autoinhibited decamer (dark grey), indicating a similar ATP-bound IstB configuration in free and STC forms. Source Data

In addition to revealing unanticipated interactions between IstB decamers, the structure of the holo-complex showed that IstB engages IstA through several contact areas (Fig. 4a). In particular, the dyad-proximal AAA+ subunits of IstB, for which nucleotide-binding sites are oriented towards IstA (monomers 1a and 1a′), interact with the catalytic monomers of the transposase (Fig. 4a and Supplementary Video 4). For IstA, this recognition occurs through the C-terminal β-barrels of the catalytic transposase subunits that are flexibly tethered to the DDE domains of the enzyme^21^. These elements dock into a deep exterior cleft formed between the N-terminal dimerization and C-terminal ATPase domains of IstB (Fig. 4b and Supplementary Video 4). Corroborating this observation, mutating residues such as Tyr343 and Arg345, which interact with IstB but are solvent exposed in the upper (non-catalytic) IstA monomers, substantially reduced the ability of the transposase to both stimulate IstB ATPase activity and be activated by IstB to promote DNA integration (Fig. 4c,d and Extended Data Fig. 7b). Similar results were obtained with an IstA(1–287) mutant, in which there was complete truncation of the β-barrel and extended C terminus of IstA.

**Extended Data Fig. 8:**
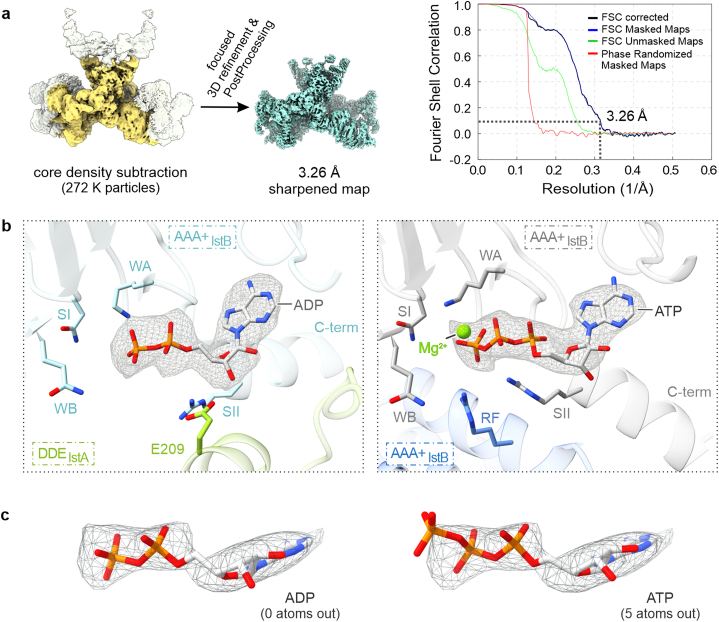
Particle subtractions and focused refinement of the holo-transpososome core. **a**, Image processing workflow of the holo-transpososome focused refinement. In gold is highlighted, in the unsharpened map, the area around the core used for the density subtraction. The map used in the modeling is shown with cyan surface. The Fourier Shell Correlation (FSC) of the sharpened cryo-EM density map is shown at the right. The contoured density thresholds are 0.00228 and 0.00525 of unsharpened and sharpened maps, respectively. **b**, Close-up view of the nucleotide binding site of monomers 1a and 1a’ (left) shows that, as opposed to the other subunits (1b, bound to ATP, shown on the right for comparison), the IstB subunits that interact with the transposase are bound to ADP. Key elements of the AAA+ pockets (SI, sensor I; SII, sensor II; WA, Walker A; WB, Walker B; RF, Arg Finger) are represented as sticks and the rest of the model as transparent cartoon. The C-terminal (C-term) α-helices that contain the sensor II (SII) are differently positioned in both ATP- and ADP- bound IstB. Density is contoured with a threshold of 0.01 in both views. **c**, Fitting of ADP (left) and ATP (right) in the nucleotide density found in the IstA-interacting IstB monomers (1a and 1a’), contoured at 0.011. The number of atoms out the density is indicated in each case.

The DDE domains of IstA also interact with the AAA+ folds of IstB through the most dyad-proximal ATPase subunits (protomers 1a and 1a′) (Fig. 4a,b,e). Close inspection of these IstB active sites revealed that the density of the nucleotide associated with these subunits (1a and 1a′) seemed different from that seen for the more internal IstB dimers that associate with ATP. We therefore performed both particle subtraction (to remove the flexible parts of the transposase and the AAA+ oligomers) and focused refinement of the holo-transpososome core; the resultant 3.2 Å reconstruction of IstA-bound IstB revealed that the density in these active sites is most consistent with ADP (Fig. 4e and Extended Data Fig. 8). In addition, a significant number of contacts were seen between an edge-helix on the DDE fold of IstA (residues 205–214) and the nucleotide-binding pocket of IstB (Fig. 4e). Mutation of a subset of these residues (for example, Glu209, which seems to engage the 3′ OH of the bound nucleotide, and Tyr213, which stabilizes the sugar and base) to alanine had a direct, negative effect on integration activity (Fig. 4d and Extended Data Fig. 7b). These interactions seemed to trigger extensive remodelling of the IstB AAA+ active site. Notably, a conserved tyrosine (Tyr170) of IstB, which in the internal decamer ATPase sites (that is, those formed between two consecutive IstB molecules) interacts with the conserved sensor I amino acid (Asn199) and the mobility of which is restrained when next to an adjacent IstB subunit, now adopts a different configuration in the IstA-engaged terminal monomer (Fig. 4e). It has been proposed that Tyr170 could act as a switch to control IstB ATPase activity, playing a part analogous to the Arg-coupler present in the loading factors of DNA polymerase clamps and the eukaryotic and prokaryotic replicative helicases^41^. Consistent with this proposal, a Y170A mutant maintained an integration activity similar to that of the wild-type protein (Fig. 4d and Extended Data Fig. 7a) while reducing nucleotide turnover (Fig. 4c). This result indicated that mutation of this residue uncouples ATP hydrolysis from the integration reaction. In addition, an α-helix that contains the conserved sensor II residue (Arg237) swivels away from the active site when IstA is bound, adopting a disengaged configuration frequently seen in product (ADP)-bound AAA+ active sites^42,43^ (Fig. 4e). Overall, these results indicate that the IstB monomers that interact with the transposase occupy a post-hydrolysis conformation (Fig. 4e and Extended Data Fig. 8).

## IstB activates the IstA transposase

**Fig. 5:**
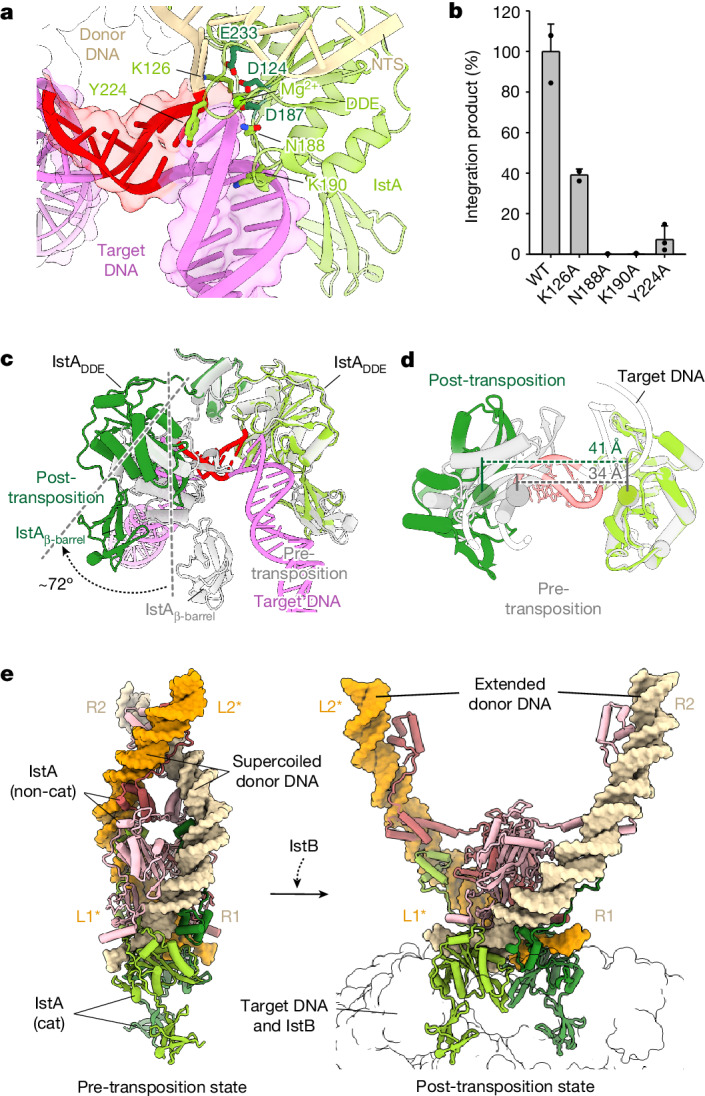
The interaction of IstA with IstB and the target DNA triggers a large conformational change in the transposase. **a**, IstA catalytic subunit (green) interacts with target DNA (violet) in the holo-transpososome. Residues interacting with DNA are shown as light green sticks, whereas the DDE motif amino acids (D124, D187, E233) are dark green sticks. Magnesium ion coordinated by the DDE motif is depicted as a light green sphere. Donor DNA transferred and NTS in dark and light wheat, respectively, and the insertion site is in red. **b**, Alanine mutants of IstA implicated in target DNA binding show degraded integration activity. Data are mean ± s.d. (*n* = 3 independent replicates). Dots indicate individual values. **c**, Comparison with IstA pre-transposition state (PDB identifier 8B4H)^21^ shows that holo-transpososome formation triggers catalytic subunit rotation. **d**, Superposition of IstA in a cleaved donor complex (grey; PDB identifier 8B4H)^21^ with a catalytic IstA subunit in the STC holo-transpososome spreads catalytic motifs (DDE triads, spheres) apart because of the IstB and target DNA interaction. **e**, Comparison between pre-transposition (PDB identifier 8B4H)^21^ and post-transposition IstA shows that catalytic monomer rotation (IstA(cat)) amplifies in non-catalytic subunits (IstA(non-cat)), forming an extended complex. Asterisks indicate that both 3D structures were determined using two R-TIRs. Source Data

**Extended Data Fig. 9:**
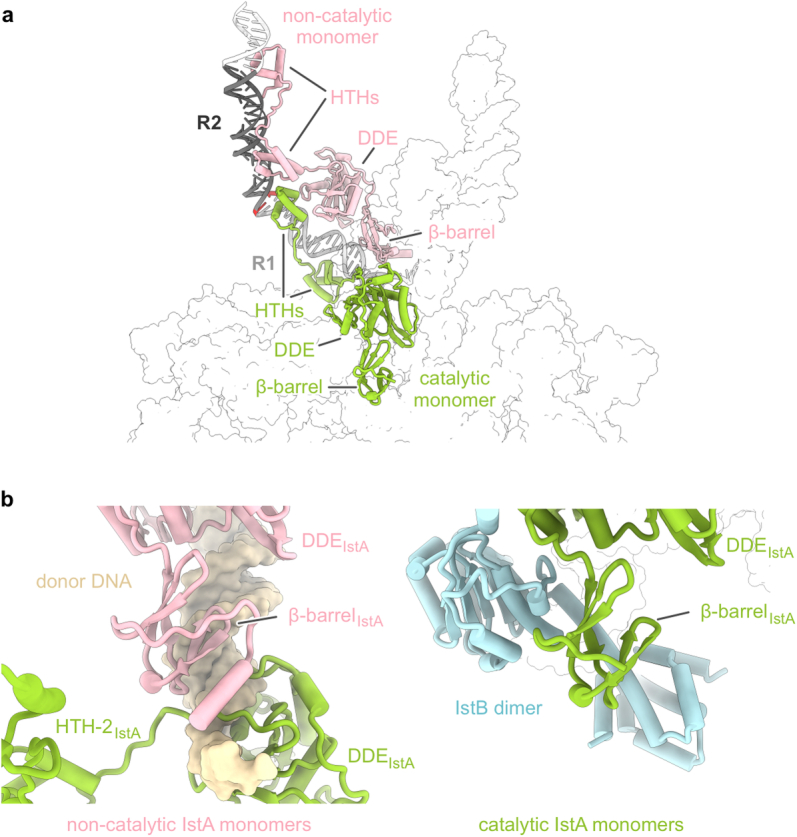
IstA interaction with the donor DNA. **a**, The two catalytic monomers of the IstA tetramer (lower chains, green) interact with the R1 repeat or a TIR while the upper structural subunits (pink) engage the R2 sequence. Only two IstA molecules and one TIR duplex are depicted, the rest of the complex is shown as a transparent surface for clarity. The base pair separating both repeats is colored in red. **b**, The β-barrel of the upper (pink) and lower (green) IstA subunits use similar areas to interact with other subunits of the transposase and the donor DNA, and IstB, respectively. The β-barrel of the upper monomers contains an extra, ordered alpha-helix (Phe361-Ala373) at the C-terminus that contacts the 5’ overhang (non-transferred strand) of the donor DNA.

In its pre-transposition state, IstA self-assembles into a highly intertwined tetramer in which each monomer interacts with all partner subunits to engage two donor DNA molecules in a right-handed (negatively) supercoiled configuration^21^. The cryo-EM reconstruction determined here showed that the primary contacts between the transposase and the TIRs are maintained in the holo-transpososome, with the HTH domains of the upper and lower transposase molecules engaging the R2 and R1 terminal repeats, respectively (Extended Data Fig. 9a). The β-barrels of the top-most (integration-site distal) subunits interact with the transposase and the transposon ends in a manner similar to that described for the isolated, cleaved donor–DNA complex^21^. However, the catalytic monomers use new and overlapping areas of the C-terminal domain to engage IstB in the STC, thereby highlighting the dual functional role of this region (Extended Data Fig. 9b). The DDE motifs of the catalytic subunits do not appreciably alter their contacts with the donor DNA. Instead, when IstB is present, the transposase establishes new interactions with the target duplex that involve DDE domain residues (for example, Lys126, Asn188, Lys190 and Tyr 224), which are solvent exposed in the upper monomers (Fig. 5a). Mutation of these residues to alanine moderately to severely reduced integration activity, a result that supports the importance of their observed contacts to the target molecule (Fig. 5b and Extended Data Fig. 7c). We sought to further validate the physiological relevance of the interactions that IstA establishes with the target DNA and IstB in the STC. To that end, we turned to a cell-free assay similar to those previously used for IS*21* and related transposon systems^23,44,45^. In brief, transposition activity was detected by quantitative PCR using cell extracts containing a donor plasmid with the TIR sequences and purified IstA and IstB. Except for a Y224A alteration, all mutants tested showed a detrimental effect on transposition activity (Extended Data Fig. 7d), confirming the functional importance of these contacts.

The interaction of IstA with IstB also remodels the transposase tetramer from its IstB-free state, which created a markedly different configuration for the transposase in the context of the holo-transpososome. In particular, new and specific contacts established between the β-barrels and the DDE modules of the lower-most (integration-site proximal) IstA monomers with the IstB ATPase induce a large-scale rotation of the integrase domains (72°) (Fig. 5c). This conformational change helps to reorientate the catalytic triad of the transposase into the appropriate spacing and geometry to perform transposition (Fig. 5d). It also induces a scissor-like action in the upper monomers of the transposase to disrupt protein–protein contacts formed before IstB binding, thus creating a highly extended configuration that untwists the DNA supercoil stabilized by IstA in the pre-transposition cleaved donor complex (Fig. 5e and Supplementary Video 5).

## Discussion

The findings presented here help address a long-standing question concerning how ATP-dependent molecular matchmakers control DNA transposition. Using IS*21* as a model system, our results showed that ATPase site integrity is important for the IstB regulator protein to support transposon integration catalysed by the IstA transposase (Fig. 1c,d). Nucleotide binding and hydrolysis regulate multiple IstB functions, including DNA binding, self-oligomerization, transposase recruitment and dissociation^27^. We note that the presence of different nucleotides, including ADP and ATPγS, as well as mutants of the catalytic centre of IstB, affected the ability of IstA to catalyse DNA integration (Extended Data Fig. 1b). These findings are consistent with previous studies of MuB and TnsC showing that the presence of non-hydrolysable nucleotide analogues or ATPase mutants can upregulate or downregulate DNA transposition, which is probably due to the capacity of these variants to stabilize different enzymatic states^9,12,28,31–33,45^. Notably, a lack of ATP hydrolysis has often been linked to a loss of target immunity, possibly because defects in ATPase function affect the ability of the transposase to reshuffle molecular matchmakers along DNA, an activity required for selecting appropriate insertion sites^28^. Although early work has suggested that IstB might also induce target immunity^20,46^, the details and molecular mechanism by which this process occurs await further investigation.

**Fig. 6:**
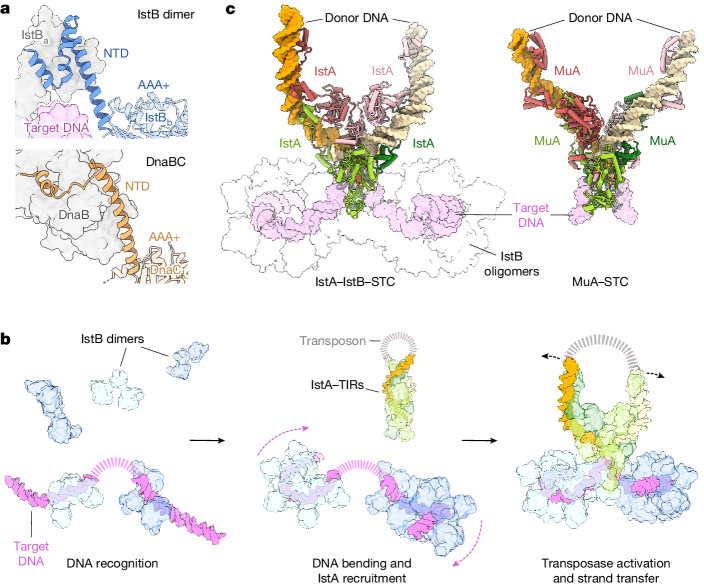
Comparison of IstAB with homologous ATPase/transposase systems and a proposed model for IstB-mediated transposition by IstA. **a**, Comparison between IstB and DnaC (PDB identifier 6QEL)^47^ N-terminal domains. **b**, Proposed stepwise model for how IstB may facilitate DNA transposition by IstA. **c**, Views of the MuA STC (PDB identifier 4FCY)^51^ and IstA–IstB STC (IstB oligomers are shown fully transparent for clarity). IstA and MuA adopt highly similar post-transpositions states.

How the nucleotide cycle of IstB is controlled by both DNA and IstA has been unclear. The high-resolution cryo-EM structure of IstB bound to DNA revealed that the N-terminal extension used by the protein for dimerization adopts an α-helical structure similar to but distinct from that found in its closest relative, the bacterial helicase loader DnaC (Figs. 1f and 6a). In the presence of ATP, each IstB dimer clasps a DNA duplex between its N-terminal and AAA+ regions, oligomerizing into a decamer that wraps the nucleic acid substrate into a 180° bend (Figs. 1e,f,i and 6b). Our reconstructions showed that the conformation of the AAA+ domains in the full-length IstB decamer is subtly different to that of a filamentous state of the isolated ATPase obtained by X-ray crystallography^27^ (Fig. 1h). This change alters the curvature of the AAA+ oligomer to subtly misalign key nucleotide-binding residues and restrain ATPase activity, similar to what has been described for DnaC when bound to its target helicase DnaB^47^. This altered conformation accounts for how an ATP-dependent transposon regulator can act as a molecular switch that is primed for catalysis after assembling on DNA, yet maintain an autorepressed configuration until after its cognate transposase is engaged.

It has been reported that like most AAA+ ATPases, oligomerized MuB and TnsC helper proteins bind a relatively straight (that is, linear) target nucleic acid segment using their central pores^9–12^. By contrast, IstB engages and bends a target DNA using an internal, U-shaped channel formed between the two pentameric halves of the decamer (Figs. 1i and 6b). Notably, for some systems (including IS*21*) it has been proposed that the transposition machinery targets S-shaped target molecules^20,40^. The configuration of the DNA in the holo-transpososome structure is fully consistent with such predictions (Fig. 3c), which suggests that the ability of individual IstB decamers to bend a target duplex may interface with local DNA flexibility or superstructure to promote insertion site scanning and selection (a similar exploratory function of the target DNA has also been suggested for MuB^48^). Consistent with this idea, previous studies have indicated that IS*21* may preferentially integrate into promoter-adjacent regions^21^, which have been described to be prone to adapt curved configurations^49^. Overall, the ability to select malleable insertion sites may be beneficial, as target DNA flexibility and deformation have proven to be crucial for numerous transposases and retroviral integrases by increasing the affinity for the target molecule and driving the directionality of the transposition reaction^36,37,48,50,51^.

In addition to selecting target DNAs, transposase helper proteins recruit their clients to suitable insertion sites (Fig. 6b). Our reconstruction of a complete IS*21* transpososome product complex revealed that the transposase is engaged by an unanticipated interaction interface between two IstB decamers (whether IstA engages the oligomers simultaneously or sequentially remains to be determined) (Fig. 4a). The decamer–decamer interface provides a means for the β-barrels of two IstA protomers to dock between the N-terminal and AAA+ domains of the dyad-proximal IstB protomers (Fig. 4b). This interaction accounts for why the IstB ATPase module is insufficient in isolation to interact with IstA^27^. Reciprocally, point mutations or truncation of the β-barrel region of IstA in turn diminishes both ATP hydrolysis by IstB and DNA integration (Fig. 4c,d). The presence of a β-barrel adjacent to a transposase DDE domain has been described for systems such as TnsB and MuA (in these proteins it is often referred to as domain IIβ), as well as for retroviral integrases^36,37,51,52^. Notably, TnsB also uses this domain to engage TnsC, albeit by interacting with a different region of the ATPase than is seen here between IstA and IstB^13^. The Tn7 transposase also utilizes its most C-terminal region, including a so-called hook element, to interact with and stimulate the ATPase activity of TnsC^13,36^. The C-terminal region of IstA is not visible in the holo-transpososome structure and, consequently, the precise role of this region during IS*21* transposition remains to be defined.

IstA is necessary to trigger IstB ATPase activity^27^, but how this stimulation occurs is not well understood. In addition to the β-barrel of IstA, the transposase engages IstB using its DDE domain. This interaction is accompanied by a remodelling of the nucleotide-binding site of the two dyad-proximal IstB subunits into what seems to be a post-hydrolysis state (Fig. 4e). Notably, nucleotide hydrolysis in AAA+ ATPases normally requires the presence of a RF residue from a neighbouring ATPase subunit. However, because there is no flanking IstB protomer to provide such an element to these two subunits (and none is evident from either adjoining IstA monomer), the ADP state captured here seems likely to arise from a rearrangement of the helper protein following the binding of IstA and the attainment of a post-transposition state. Further experiments will be needed to determine the precise configuration of IstB following transposase recruitment but before strand exchange to resolve the nucleotide-mediated reorganization events that may have led to the state imaged here.

Although the bending of a target DNA sequence can be important for transposition, many transposases and retroviral integrases are capable of remodelling the nucleic acid substrate without the need for additional factors^38,50^. Why some transposable elements have elected to use a secondary molecular switch to control strand transfer and how such factors regulate the activity of the cognate transposase has been obscure. For bacteriophage Mu, it has been proposed that MuA can adopt a pre-transposition state that is topologically consistent with that described for IstA^21,53,54^, corresponding to an intertwined inactive tetramer that synapses the two transposon ends in a supercoiled configuration^21^ (Figs. 5e and 6b). In both cases, it has been suggested that the pre-strand transfer configuration of the transposase (that is, the cleaved donor complex) is a dormant state that needs to be activated by interactions between the helper protein ATPase and transposase regions C-terminal to the catalytic DDE domain^21,28^. It therefore seems that one role of the multiple terminal repeats present in donor DNAs is to maintain the transposase in an autoinhibited configuration before binding of the helper.

Notably, the IstB-associated post-transposition state of IstA imaged here is also highly similar to one described for the MuA transposase^51^ (Fig. 6c). This congruency suggests that reorganization events seen for IstA may similarly occur in MuA and, perhaps, other transposases that depend on dedicated ATPase regulators for function. Overall, the molecular effector switch described in this work allows the transposase to remain in an inactive state (possibly to minimize potentially detrimental DNA breaks in the cell). Moreover, the function of helper ATPases is to both mark and remodel a suitably flexible insertion site (away from the transposon sequence) into a configuration compatible with strand transfer while also triggering a conformational change in the transposase to activate transposition. Future studies will be needed to establish the precise mechanistic role of nucleotide turnover by IstB and the extent to which other transposition-related molecular matchmakers use analogous approaches to activate their cognate transposases.

## Methods

### IstA purification

*G.* *stearothermophilus* full-length IstA was purified as previously described^21^. In brief, the full-length transposase sequence was expressed and purified using a pET-derived vector with a TEV-protease-cleavable MBP-His_6_ tag at the C terminus (2CcT vector) in the C41(DE3) *Escherichia coli* strain, induced after reaching an optical density (OD) at 600 nm of 0.7 with 0.5 mM β-isopropyl-d-1-thiogalactopyranoside during 4 h of incubation at 37 °C in 2×YT (yeast extract tryptone) medium supplemented with 1% glucose. Cells were resuspended in lysis buffer (50 mM HEPES pH 7.5, 750 mM NaCl, 5 mM MgCl_2_, 5% glycerol and 1 mM β-mercaptoethanol) supplemented with protease inhibitors (complete, mini protease inhibitor tablets; Roche), lysed by sonication and centrifugated (20,000*g*, 30 min, 4 °C). The soluble fraction was filtered through a 0.45 µm pore-size filter and bound to a Ni-Sepharose column (5 ml His-Trap HP Chelating; Cytiva), washed with lysis buffer containing 30 mM imidazole and eluted (with 50 mM HEPES pH 7.5, 150 mM NaCl, 5 mM MgCl_2_, 5% glycerol, 300 mM imidazole and 1 mM β-mercaptoethanol) directly onto a cationic exchange column (5 ml Hi-Trap SP HP column; Cytiva). The ionic exchange column was further washed with 50 mM HEPES pH 7.5, 300 mM NaCl, 5 mM MgCl_2_, 5% glycerol and 1 mM β-mercaptoethanol, and the sample was eluted with a salt gradient up to 1 M NaCl. After cleaving the MBP tag by incubating at 4 °C overnight with TEV protease, the sample was passed through a second histidine-affinity column. The protein was finally concentrated and injected into a preparative HiPrep Sephacryl S-200 16/60 HR column (Cytiva) equilibrated in 50 mM HEPES pH 7.5, 750 mM NaCl, 5 mM MgCl_2_, 5% glycerol and 1 mM β-mercaptoethanol, at room temperature. Purified transposase was concentrated, aliquoted and flash-frozen in liquid nitrogen and stored at −80 °C until later use.

### IstB purification

*G.* *stearothermophilus* full-length IstB was purified as previously described^27^. In brief, His_6_-MBP-IstB was expressed using a pET-derived vector in *E.* *coli* BL21codon-plus (DE3) RIL cells (Stratagene). Cells were grown at 37 °C in 2×YT medium, induced at an OD at 600 nm of 0.8 with 0.5 mM β-isopropyl-d-1-thiogalactopyranoside at 37 °C for 3.5 h, collected by centrifugation, resuspended in resuspension buffer (20 mM HEPES pH 7.5, 500 mM NaCl, 5 mM MgCl_2_, 5% glycerol, 30 mM imidazole, 1 mM β-mercaptoethanol and 0.1 mM ADP) supplemented with protease inhibitors (complete, mini protease inhibitor tablets; Roche), and lysed by sonication. The supernatant obtained after centrifugation (20,000*g*, 30 min, 4 °C) was filtered through a 0.45 μm pore-size filter (Sartorius), run over a Ni-Sepharose column (His-Trap HP, Cytiva) and then washed with resuspension buffer. The protein was eluted with a step-wise imidazole gradient. Peak fractions were pooled and run onto an amylose-affinity resin equilibrated with resuspension buffer and cleaved on-column overnight at 4 °C with PreScission-protease. After elution of the cleaved protein with resuspension buffer, the sample was concentrated with ultrafiltration devices and injected into a HiPrep Sephacryl S-200 16/60 HR gel-filtration column (Cytiva) preequilibrated in resuspension buffer at room temperature. Fractions corresponding to IstB were pooled and concentrated, aliquoted and flash-frozen in liquid nitrogen and stored at −80 °C.

### In vitro integration assay

Integration reactions were performed in 50 mM HEPES pH 7.5, 150 mM NaCl, 5 mM MgCl_2_, 5% glycerol, 0.05 mg ml^–1^ BSA and 1 mM ATP. IstA was pre-incubated with the right donor DNA (R-TIR with a 5-nt-long 5′ overhang), both present at 0.25 µM in the final reaction. IstB (2 µM final concentration) was independently pre-incubated with supercoiled plasmid pSG483 (10 nM final concentration) serving as the target DNA. Both proteins were then mixed in 30 µl and incubated for 15, 30 or 60 min at 37 °C. To stop the reactions, the mixtures were incubated for 20 min at 37 °C with proteinase K (0.25 mg ml^–1^ final concentration), SDS (1% final concentration) and EDTA (28 mM final concentration). Samples were run for 18 h on 1.5% (w/v) Tris–acetate–EDTA (TAE) agarose gels (40 mM sodium acetate, 50 mM Tris–HCl pH 7.9 and 1 mM EDTA) at 2–2.5 V cm^–1^. To visualize the DNA, gels were stained with 0.5 μg ml^–1^ ethidium bromide in TAE buffer for 20 min, destained in TAE buffer for 10 min and exposed to UV transillumination. DNA bands were quantified using Image Lab software (v.5.2.1, Bio-Rad).

### Cell-free transposition assay

Cells extract was obtained as previously described^23^. In brief, *E.* *coli* BL21 (DE3) cells were grown until reaching an OD at 600 nm of 0.6, collected by centrifugation and resuspended in 25 mM HEPES pH 7.5, 2 mM DTT and 100 mM KCl. Cells were treated with 250 μg ml^–1^ lysozyme for 20 min and 10 mM MgCl_2_ for 30 min. Thereafter, cells were frozen in liquid N_2_ and lysed by thawing on ice. Cell debris was removed by centrifugation at 14,000 r.p.m. for 30 min. Transposition reactions were carried out in 20 μl final volume containing 16 μl of reaction buffer (25 mM HEPES pH 7.5, 50 mM KCl, 10 mM MgCl_2_, 1 mM DTT, 50 μg ml^–1^ BSA, 150 μM dNTPs and 1 mM ATP), 1 μM IstA, 4 μM IstB, 50 ng of donor plasmid (1B-LIC vector containing lacZ flanking by IS*21* TIRs) and 1 μl of cell extract. Reactions were incubated at 37 °C for 60 min and the frequency of insertions relative to the control without proteins was determined by quantitative PCR with a set of primers corresponding to a specific region of the donor plasmid (primer forward: 5′-TGTAATTCAGCTCCGCCATC-3′, primer reverse: 5′-GGTGTCTCTTATCAGACCGTTTC-3′) and a set of primers corresponding to the transposition product (primer forward: 5′-CGATTACTGCATCATTCCATCATTT-3′, primer reverse: 5′-AGGACCTTTCATTGATCCTTCTG-3′). Each mixture (10 μl) contained 1 μl of transposition reaction, 500 nM forward primer, 500 nM reverse primer and 1× SYBR. Fluorescence was measured using a LightCycler 96 Instrument (Roche). Data were analysed using the 2^–ΔΔ*C*t^ method normalizing with donor plasmid *C*t.

### ATPase assays

Reactions (50 µl) were set up containing 50 mM HEPES pH 7.5, 150 mM NaCl, 10 mM MgCl_2_ and 1 mM ATP. IstB (5 µM) was then added either alone or in combination with 0.5 µM IstA. After 1 h of incubation at 37 °C, ATPase activity was measured using a PiColorLock Phosphate Detection system (Abcam). Plates were scanned in a Varioskan LUX microplate reader (Thermo Scientific).

### STC DNA reconstitution

To reconstitute the STC DNA, we designed a construct that mimics the transposition product^36–38,55^. The construct was obtained by annealing three single-stranded DNA molecules (Fig. 2b and Extended Data Fig. 4). The longest strand (TIR-transferred strand; 130 nt) contains the complete sequence of the right transposon end (including the two R1 and R2 repeats), the target DNA and the insertion site. The other two DNA molecules contain the sequences complementary to the donor (non-transferred strand; 60 nt) and target (target-reverse complement; 70 nt) DNAs. The individual oligonucleotides (Integrated DNA Technologies) were resuspended in 20 mM HEPES pH 7.5 and 50 mM NaCl, mixed in equimolar concentrations, heated up to 95 °C for 5 min and gradually cooled down to 10 °C over 10 h (Extended Data Fig. 4).

### IstB–target DNA complex formation and vitrification

To obtain the complex of IstB bound to a target DNA, wild-type IstB was mixed with a random 60-mer duplex (5′-TGCTTGCGATGATCCGACGTGTTAGCCACGCTGACTAGTTATGCCATGCCTCCCTTCAGG-3′) with a 6.5:1 molar ratio and dialysed overnight at 4 °C against 20 mM HEPES pH 7.5, 150 mM NaCl, 5 mM MgCl_2_, 5% glycerol, 1 mM ATP and 1 mM DTT. The sample was then loaded onto a Superdex 200 Increase 5/150 GL equilibrated in dialysis buffer, at room temperature. The fractions corresponding to the peak containing the IstB–DNA complex were aliquoted, flash-frozen with liquid nitrogen and stored at −80 °C.

For the cryo-EM experiments, the sample was diluted in 20 mM HEPES pH 7.5, 150 mM NaCl, 5 mM MgCl_2_, 1 mM ATP, 1 mM DTT and 0.015% NP-40 (to try to favour the formation of a thin and uniform ice layer). Next, 3 μl of the IstB–DNA complex (about 2 μM) was applied to glow-discharged Quantifoil Gold 2:1, 300 mesh grids with a home-made continuous carbon coating, blotted for 4 s (blot force of +25) and frozen in liquid ethane using a Vitrobot Mark IV plunging system (Thermo Fisher Scientific).

### IstA–IstB–STC holo-transpososome complex formation

To obtain the holo-transpososome complex, numerous buffer conditions and protein-to-DNA stoichiometries were initially tested using gel filtration chromatography and negative-staining EM. Finally, IstA was mixed with the STC DNA in a 3.3:1 (protein to DNA) molar ratio and diluted in STC buffer (20 mM HEPES pH 7.5, 150 mM NaCl, 5 mM MgCl_2_, 5% glycerol, 1 mM ATP and 1 mM DTT) until IstA was present at 2.4 μM. The sample was then incubated for 45 min at 37 °C, mixed with 12 μM IstB_E167Q_ in STC buffer (IstB to IstA final 5:1 molar ratio), and incubated for 30 min at 37 °C.

For negative-stain EM, the holo-transpososome was further diluted in STC buffer. Next, 4 μl (1 μM IstB referred to IstB monomer) of the sample was applied to a glow-discharged continuous carbon-coated grid (Electron Microscopy Sciences), incubated for 1 min and washed twice in 50 μl drops of MilliQ water before being incubated for 1 min in 2 × 10 μl droplets of 2% uranyl acetate stain. Images were collected using a JEOL 1230 microscope equipped with a TemCam-F416 camera (TVIPS), at a magnification of ×40,000, corresponding to a pixel size of 2.8 Å per pixel. They were imported to RELION-4.0 (refs. ^56,57^) and the contrast transfer function (CTF) was estimated using CTFFIND (v.4.1)^58^. Micrographs were picked using Topaz and subjected to 2D classification using RELION-4.0 (refs. ^56,57,59^).

For cryo-EM grid preparation, the sample was diluted in 20 mM HEPES pH 7.5, 150 mM NaCl, 5 mM MgCl_2_, 1 mM ATP, 1 mM DTT and 0.015% NP-40. Next, 3 μl of the transpososome complex (1.5 μM IstB referred to IstB monomer) was applied to glow-discharged Quantifoil Gold 2:1, 300 mesh grids coated with a second layer of homemade thin continuous carbon. After 1 min of incubation, grids were blotted for 4 s (blot force of +25) and frozen in liquid ethane using a Vitrobot Mark IV (Thermo Fisher Scientific).

### Cryo-EM data collection, image processing and atomic model building of IstB–target–DNA complex

Cryo-EM grids were pre-screened in a JEOL 1230 microscope and in a FEI Talos Artica microscope equipped with a TemCam-F416 (TVIPS) and a Falcon III camera, respectively (Thermo Fisher Scientific). High-resolution data of the IstB complex with target DNA were collected on a Titan Krios electron microscope operated at 300 kV (Diamond Light Source). Imaging was performed with EPU at a nominal magnification of ×81,000 (calibrated physical pixel size of 1.06 Å per pixel; super-resolution of 0.53 Å per pixel) using a Gatan K3 BioQuantum direct electron detector operating in super-resolution counting mode. The nominal defocus range for the dataset extended from −1.2 μm to −2.7 μm in 0.3 μm increments. Each movie was recorded during 5 s and fractioned in 50 frames. The dose rate was 1.2 e^–^ per Å^2^ per frame, resulting in an accumulated exposure of 60 e^–^ Å^–2^ (Extended Data Table 1).

A total of 6,214 movies were imported into RELION-3.0 (refs. ^57,60^), motion-corrected and electron-dose-weighted with MOTIONCOR2 (ref. ^61^) (Extended Data Fig. 2). The CTF was estimated using GCTF^62^. They were manually curated to remove patchy or cristaline ice, obtaining a subset of 3,812 micrographs. A subset of the micrographs was picked with CRYOLO^63^, binned by two and subjected to 2D classification. The resulting 2D averages were then used as templates to pick the entire dataset with RELION-3.0 (ref. ^57^). A total of 2,439,865 particles were extracted and downsampled to 2.12 Å per pixel. After 2D classification, 1,451,514 particles were selected and subjected to 3D classification using C1 symmetry, which separated full pentamers of dimers from incomplete, tetrameric complexes. The 306,745 particles from the best class were extracted with the original pixel size of 1.06 Å per pixel and used as input for a subsequent 3D refinement, run using C2 symmetry and a soft-edged mask that followed the contour of the particle, which resulted in a 3.35 Å resolution map. The particles where subjected to CTF refinement and Bayesian polishing, generating the final 3.2 Å resolution map.

To improve the density corresponding to IstB N-terminal domains, the dataset was subjected to focused 3D classification (Extended Data Fig. 2g,h). First, symmetry expansion was applied to the final 306,745 particles. A mask was then created around the N-terminal domains of the second dimer using Chimera (v.1.14)^64^. This particular dimer was selected because of its central location and, therefore, probably more rigid configuration. The density of the remaining decamer was subtracted from the particles and the selected N-terminal domains were subjected to a round of 3D classification without re-aligning the particles (C1 symmetry, *T* = 1,000). One of the resulting maps, which showed continuous and clear density for the polypeptide chain, was used for modelling this region.

A previously determined crystal structure of the IstB ATPase domain (PDB identifier 5BQ5)^27^ was rigid-body docked into one of the monomers of the cryo-EM density map obtained from the 3D refinement. The N-terminal domain was manually de novo built using both the map of the full decamer and that obtained from the focused classification as references. The complete monomer was then subjected to a round of model building and real space refinement with COOT and PHENIX-1.19 using Ramachandran, rotamer, geometry and secondary structure restraints^65,66^. The refined monomer was then docked into the four remaining positions to generate the asymmetric unit and subjected to an additional round of model building and real space refinement. The full decamer was obtained applying C2 symmetry operators to the refined unit. IstB has no clear sequence specificity and the density for the nucleic acid appeared to be less defined than the protein probably as a consequence of DNA sliding. The density, however, clearly showed the position of the characteristic mayor and minor grooves. iMODFIT (v.1.2) was used to generate a curved DNA duplex that followed the density map^67^. The complete model was obtained after some final interactive rounds of real space refinement and validation with PHENIX and MOLPROBITY using Ramachandran, rotamer, geometry, secondary structure, base planarity and base stacking restraints and NCS constraints.

### Cryo-EM data collection, image processing and atomic modelling of the IstA–IstB–STC holo-transpososome complex

Cryo-EM grids were screened in a JEOL 1230 microscope and in a FEI Talos Artica microscope equipped with a TemCam-F416 (TVIPS) and a Falcon III camera, respectively (Thermo Fisher Scientific). High-resolution data of the holo-transpososome complex were collected from two grids (obtained under identical biochemical and vitrification conditions) on a Titan Krios electron microscope operated at 300 kV (BREM Biofisika, Bilbao). Imaging was performed using EPU at a nominal magnification of ×105,000 (calibrated physical pixel size of 0.82 Å per pixel; super-resolution of 0.41 Å per pixel) with a Gatan K3 BioQuantum direct electron detector operating in super-resolution counting mode. Each movie was recorded during 2.1 s in 50 frames with a nominal defocus range of −1 μm to −2.6 μm (increments of 0.3 μm). The dose rate was 1 e^−^ Å^–2^ per frame, resulting in an accumulated exposure of 51.3 e^−^ Å^–2^ (Extended Data Table 1). Overall, 15,206 and 21,070 movies were acquired from the two grids.

The movies from each grid were initially pre-processed independently. They were imported into RELION-4.0 (refs. ^56,57^), motion-corrected and electron-dose-weighted with MOTIONCOR2 (ref. ^61^), and the CTF was estimated using CTFFIND (v.4.1)^58^. Micrographs were picked using Topaz^59^. From the first grid, a total of 1,395,290 particles were extracted and downsampled to 1.98 Å per pixel (Extended Data Fig. 5). After 2D classification, 167,791 particles were selected, re-extracted with a pixel size of 0.98 Å per pixel and used as input for a subsequent 3D classification using a soft-edged mask. The initial 3D classification, using C1 symmetry, identified various oligomeric states of IstB in some of the models that seemed to result from the detachment of IstA-distant flexible dimers (in line with findings from the isolated ATPase complexed with target DNA). However, the three most IstA-proximal IstB dimers from each oligomer consistently exhibited greater rigidity and homogeneity. Consequently, using C2 symmetry for 3D classification and subsequent refinement steps produced similar results, with enhanced resolution and map quality in the central region of the complex. The 140,441 particles from the best classes were then subjected to 3D refinement, run using a soft-edged mask that followed the contour of the particle, and subjected to CTF refinement and Bayesian polishing that allowed us to generate a 4.3 Å resolution map. From the second grid, a total of 2,707,350 particles were extracted and downsampled to 1.98 Å per pixel. After 2D classification, 212,669 particles were selected and subjected to 3D refinement with a soft-edged mask. They were then re-extracted with a pixel size of 0.98 Å per pixel and subjected to CTF refinement and Bayesian polishing, resulting in a 3.65 Å resolution map. The CTF-refined and Bayesian-polished particles from both grids were then pooled together (353,110 total particles) and used as input for a subsequent 3D classification, using C2 symmetry and a tight mask. Overall, 272,218 particles from the best classes were used for a last round of 3D refinement, using C2 symmetry and a soft-edged mask, to obtain a 3.62 Å resolution map.

To improve the density map around relevant elements, a focused 3D refinement of the core (removing the flexible regions) was performed using RELION-4.0 (refs. ^56,57^). First, a soft-edged mask created following the contour of the core was used to do a particle subtraction on the 272,218 particles. Subsequently, a focused 3D-refinement (C2 symmetry) produced an improved EM density at 3.26 Å resolution (Extended Data Fig. 8).

Monomers of IstA (PDB identifier 8B4H)^21^ and IstB (obtained previously in the complex of IstB–target DNA) were initially fitted as a rigid body into the unsharpened maps and later manually modelled in COOT^65^ using the sharpened maps for the fitting of the lateral chains. The asymmetric unit of the complex, which contained two monomers of IstA, ten molecules of IstB and three strands of DNA (chains A and C, chains E to N and chains a, b and c, respectively) was improved by alternating rounds of model building and real space refinement with COOT and PHENIX (v.1.20)^65,66^, applying rotamer, Ramachandran, secondary structure and geometry restraints for the protein, and stacking, hydrogen bonds and base-pair parallel planes restraints for the DNA (generated using LIBG and ProSMART tools from CCP4 package)^68^. The complete model was generated by imposing C2 symmetry operators to the asymmetric unit, resulting in a macromolecular assembly composed by four chains of IstA (A–D), 20 monomers of IstB (E–X) and 6 strands of DNA (a–f). The STC was generated as duplex DNA using GRAPHITE-LIFE EXPLORER^69^. The symmetrized model was subjected to interactive rounds of real space refinement, applying NCS constraints, and validation performed with PHENIX-1.20, COOT, MolProbity and the PDB validation tool (OneDep: https://validate-rcsb-1.wwpdb.org)^65,66^. Figures were generated using Chimera (v.1.15) and ChimeraX (v.1.5)^64,70^.

### Reporting summary

Further information on research design is available in the Nature Portfolio Reporting Summary linked to this article.

## Online content

Any methods, additional references, Nature Portfolio reporting summaries, source data, extended data, supplementary information, acknowledgements, peer review information; details of author contributions and competing interests; and statements of data and code availability are available at 10.1038/s41586-024-07550-6.

### Supplementary information

Supplementary Information Supplementary Figs. 1 and 2 and Supplementary Table 1.

Reporting Summary

Supplementary Video 1 **3D organization of the complex of IstB–target DNA**. The video shows how IstB uses its N-terminal domains to dimerize and how it forms a pentamer of dimers mediated by AAA+ contacts that traps and bends the target DNA.

Supplementary Video 2 **Molecular organization of the IstA–IstB–STC holo-transpososome**. The structure is depicted following the colour convention used in Fig. 2.

Supplementary Video 3 **Close-up view of the contacts between IstB oligomers in the IS*21*** **holo-transpososome**. The interacting residues are displayed as gold surface in the context of the holo-transpososome.

Supplementary Video 4 **Details of the interaction between IstA and IstB**. The interaction of the β-barrels and catalytic domains of two IstA subunits with IstB is shown in detail and in the wider context of the full transpososome.

Supplementary Video 5 **Conformational change of the transposase IstA and the donor DNA from the pre-transcription (PDB ID: 8B4H) to the post-transposition state**. The video shows a morphing between the pre-transposition closed intertwined plectoneme formed by IstA (PDB ID: 8B4H) and the open configuration adopted in the transpososome.

### Source data

Source Data Fig. 1

Source Data Fig. 3

Source Data Fig. 4

Source Data Fig. 5

Source Data Extended Data Fig. 7

## Figures and Tables

**Extended Data Table 1:**
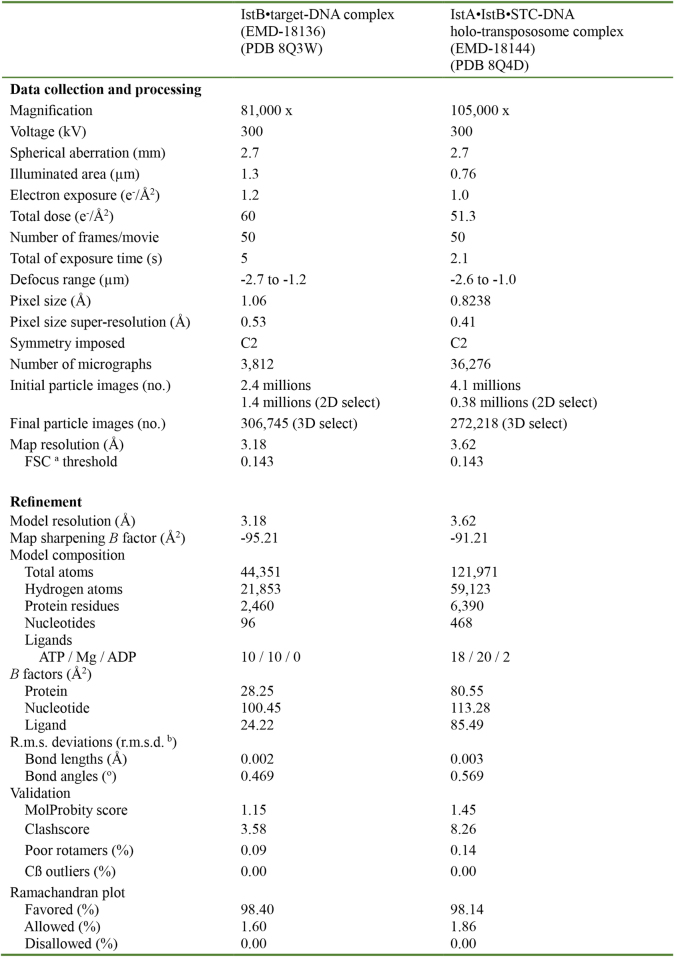
Cryo-EM data collection and refinement statistics

^a^FSC; Fourier Shell Correlation. ^b^r.m.s.d.; root mean square deviation.

## Data Availability

The cryo-EM densities and atomic coordinates of the structures obtained in this study have been deposited into the Electron Microscopy Data Bank and PDB, respectively, with the following accession codes: EMD-18136 and PDB 8Q3W for IstB–target DNA; and EMD-18144 and PDB 8Q4D for the IstA–IstB–STC holo-transpososome. PDB codes of previously determined structures used in this manuscript are as follows: 5BQ5 (IstB AAA+ domains), 8B4H (IstA pre-cleaved donor complex), 6QEL (DnaBC complex) and 4FCY (MuA). Source data are provided with this paper.
