# Supplementary material for: Molecular basis for transposase activation by a dedicated AAA+ ATPase
Source: Nature. 2024 Jun 26;630(8018):1003–11. doi: 10.1038/s41586-024-07550-6 (PMC11208146; doi:10.1038/s41586-024-07550-6)
Supplement: Supplementary file 1 — Supplementary Figs. 1 and 2 and Supplementary Table 1. [file 41586_2024_7550_MOESM1_ESM.pdf]

nature portfolio

https://doi.org/10.1038/s41586-024-07550-6

Supplementary information

Molecular basis for transposase activation by a dedicated AAA+ ATPase

In the format provided by the authors and unedited

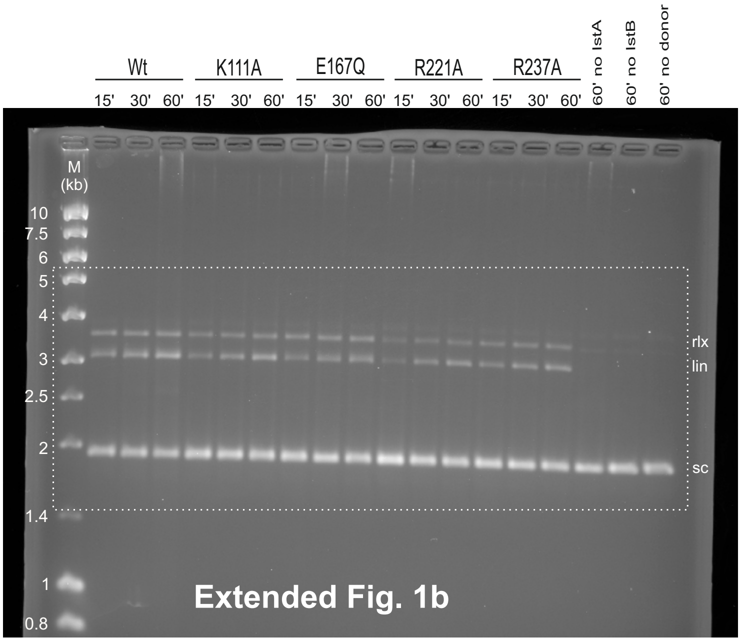
Wt
K111A
E167Q
R221A
R237A
15' 30' 60' 15' 30' 60' 15' 30' 60' 15' 30' 60' 15' 30' 60'
60' no IstA
60' no IstB
60' no donor
M (kb)
10
7.5
6
5
4
3
2.5
2
1.4
1
0.8
rlx
lin
sc
Extended Fig. 1b

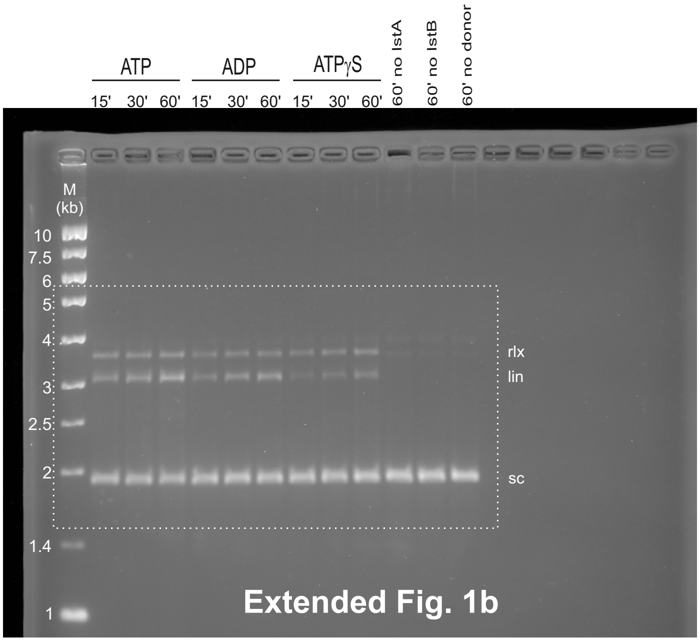
ATP
ADP
ATPγS
15' 30' 60' 15' 30' 60' 15' 30' 60'
60' no IstA
60' no IstB
60' no donor
M (kb)
10
7.5
6
5
4
3
2.5
2
1.4
1
rlx
lin
sc
Extended Fig. 1b

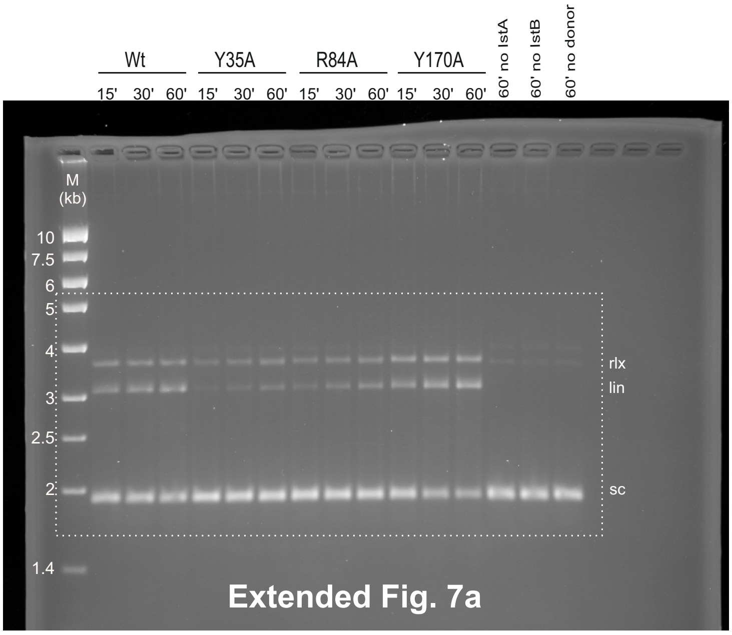
Wt
Y35A
R84A
Y170A
15' 30' 60' 15' 30' 60' 15' 30' 60' 15' 30' 60'
60' no IstA
60' no IstB
60' no donor
M (kb)
10
7.5
6
5
4
3
2.5
2
1.4
rlx
lin
sc
Extended Fig. 7a

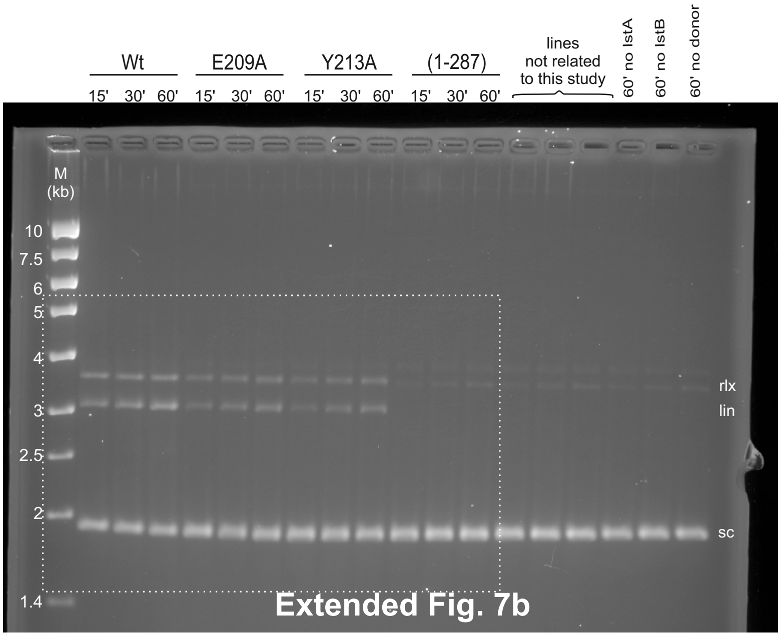
Wt
E209A
Y213A
(1-287)
15' 30' 60' 15' 30' 60' 15' 30' 60' 15' 30' 60'
lines not related to this study
60' no IstA
60' no IstB
60' no donor
M (kb)
10
7.5
6
5
4
3
2.5
2
1.4
rlx
lin
sc
Extended Fig. 7b

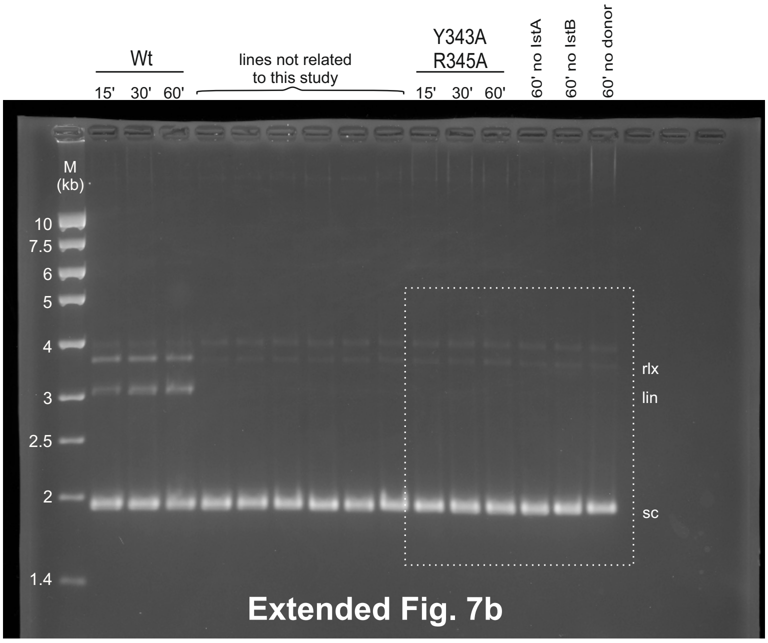
Wt
15' 30' 60'
lines not related to this study
Y343A R345A
15' 30' 60'
60' no IstA
60' no IstB
60' no donor
M (kb)
10
7.5
6
5
4
3
2.5
2
1.4
rlx
lin
sc
Extended Fig. 7b

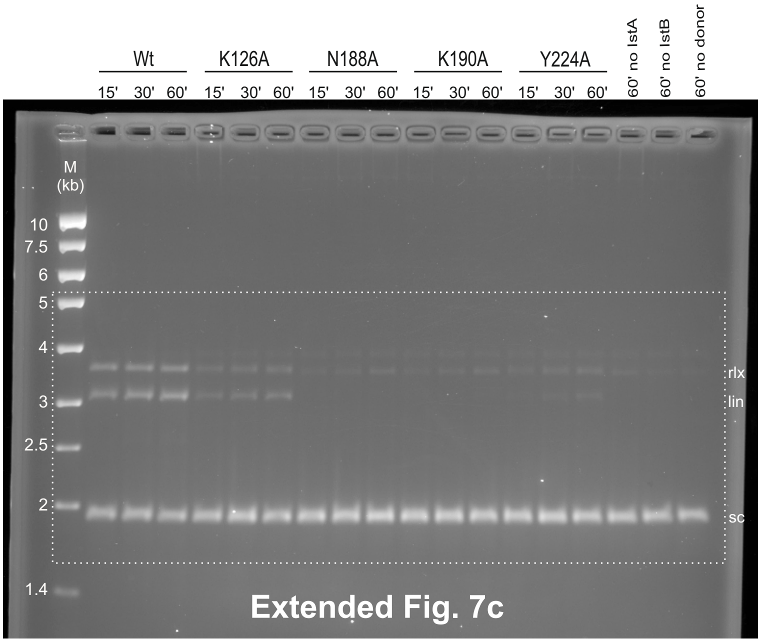
Wt
K126A
N188A
K190A
Y224A
15' 30' 60' 15' 30' 60' 15' 30' 60' 15' 30' 60' 15' 30' 60'
60' no IstA
60' no IstB
60' no donor
M (kb)
10
7.5
6
5
4
3
2.5
2
1.4
rlx
lin
sc
Extended Fig. 7c

Replicates

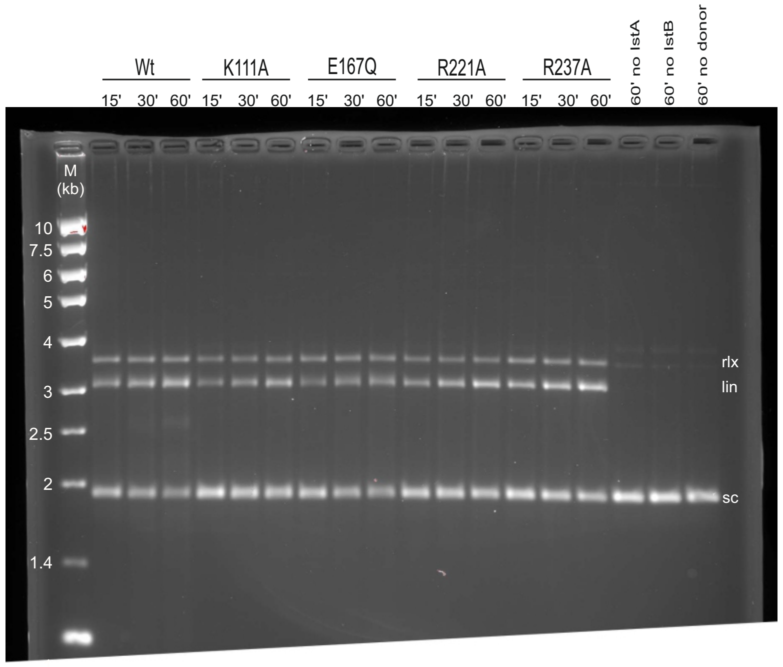
Wt
K111A
E167Q
R221A
R237A
15' 30' 60' 15' 30' 60' 15' 30' 60' 15' 30' 60' 15' 30' 60'
60' no IstA
60' no IstB
60' no donor
M
(kb)
10
7.5
6
5
4
3
2.5
2
1.4
rlx
lin
sc

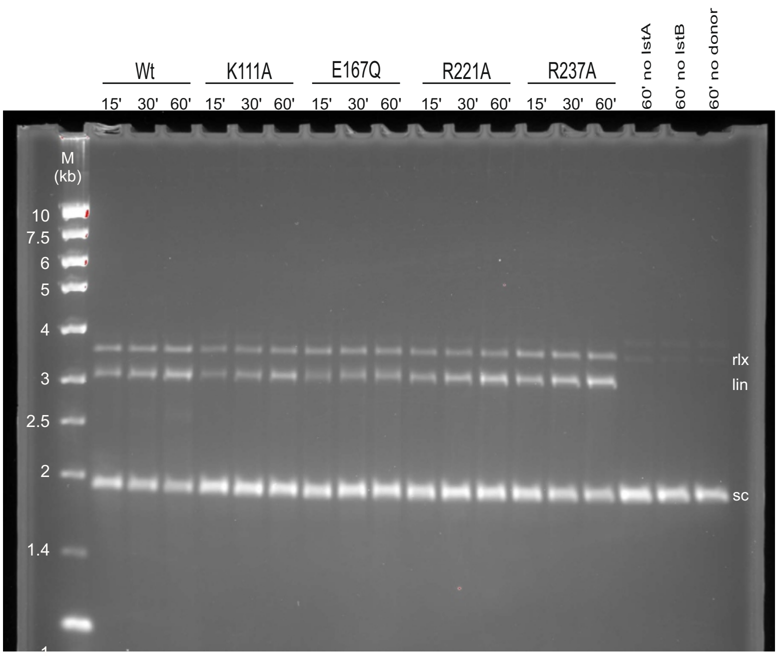
Wt
K111A
E167Q
R221A
R237A
15' 30' 60' 15' 30' 60' 15' 30' 60' 15' 30' 60' 15' 30' 60'
60' no IstA
60' no IstB
60' no donor
M
(kb)
10
7.5
6
5
4
3
2.5
2
1.4
rlx
lin
sc

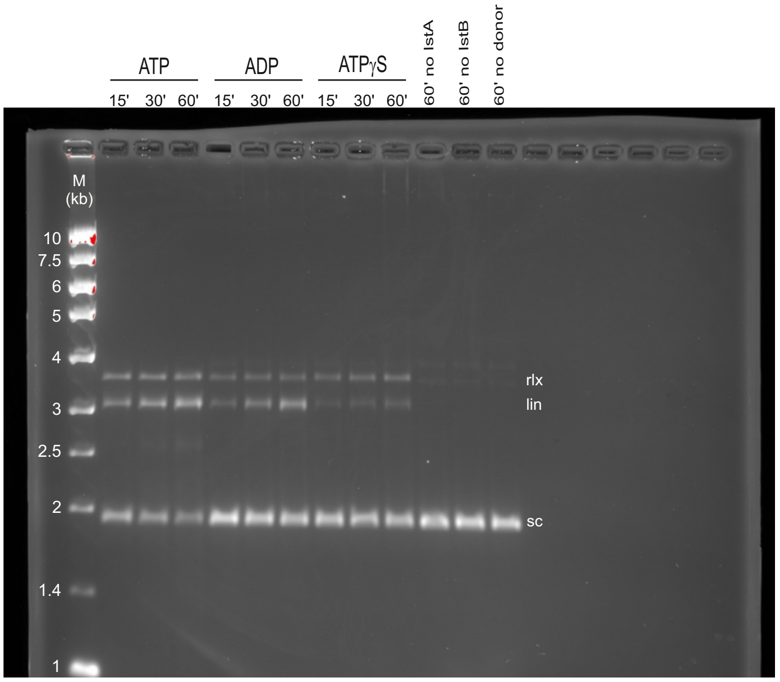
ATP
ADP
ATPγS
15' 30' 60' 15' 30' 60' 15' 30' 60'
60' no IstA
60' no IstB
60' no donor
M
(kb)
10
7.5
6
5
4
3
2.5
2
1.4
1
rlx
lin
sc

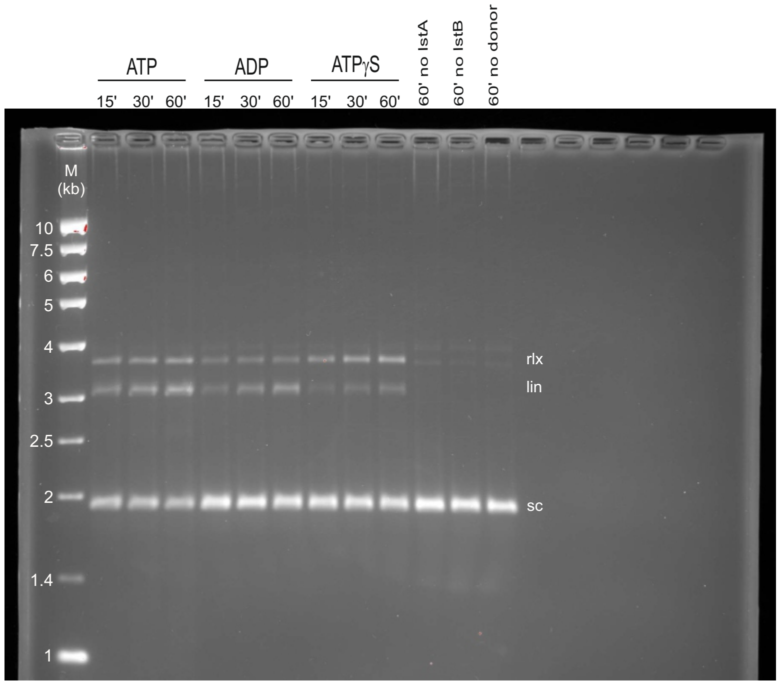
ATP
ADP
ATPγS
15' 30' 60' 15' 30' 60' 15' 30' 60'
60' no IstA
60' no IstB
60' no donor
M
(kb)
10
7.5
6
5
4
3
2.5
2
1.4
1
rlx
lin
sc

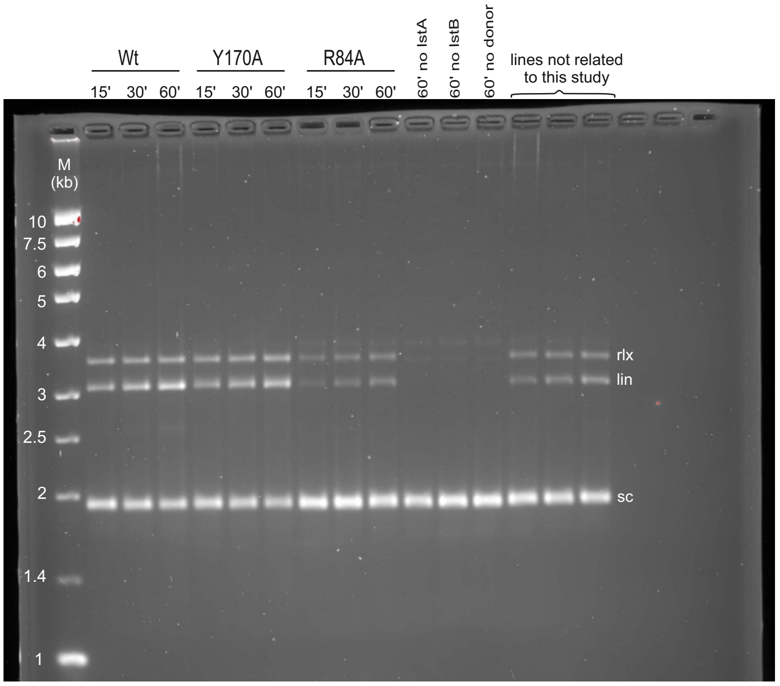
Wt
Y170A
R84A
15' 30' 60' 15' 30' 60' 15' 30' 60'
60' no IstA
60' no IstB
60' no donor
lines not related
to this study
M
(kb)
10
7.5
6
5
4
3
2.5
2
1.4
1
rlx
lin
sc

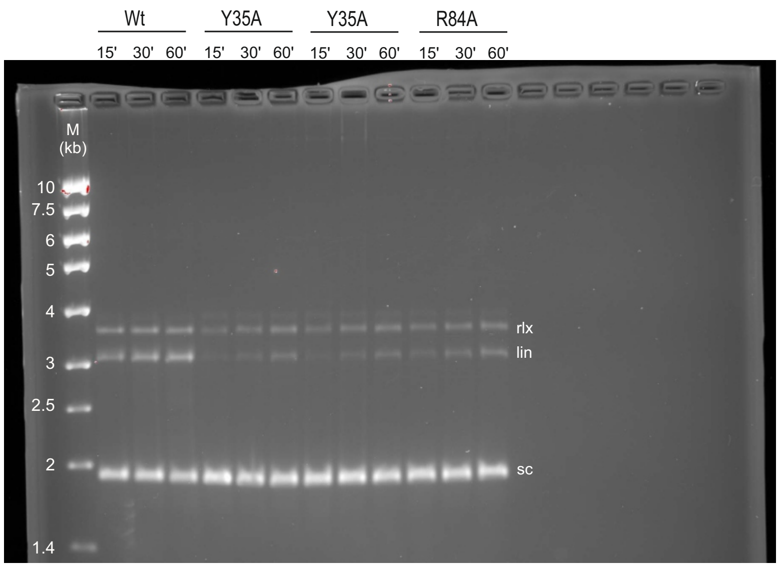
Wt
Y35A
Y35A
R84A
15' 30' 60' 15' 30' 60' 15' 30' 60' 15' 30' 60'
M
(kb)
10
7.5
6
5
4
3
2.5
2
1.4
rlx
lin
sc

Replicates

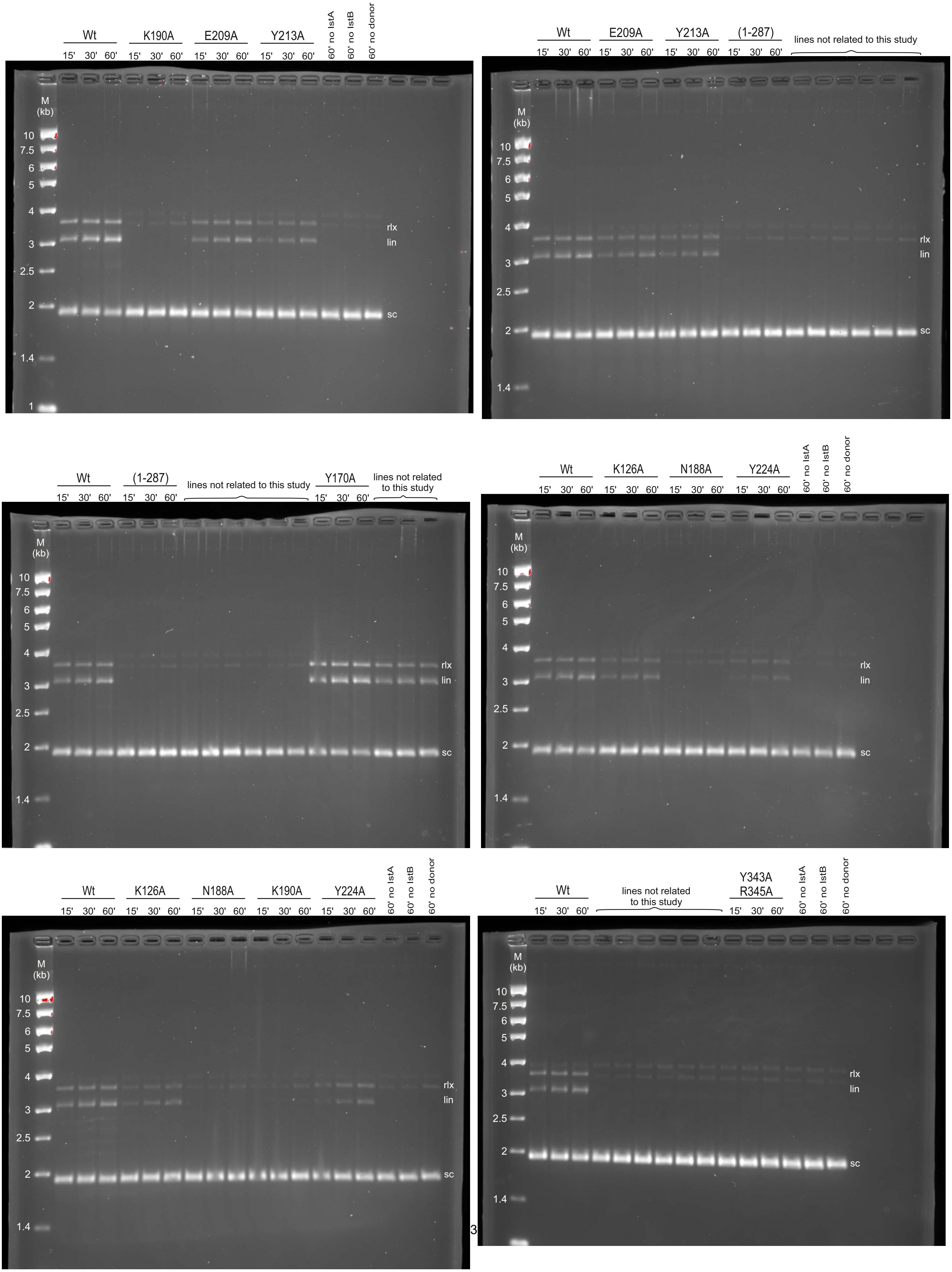
Wt
K190A
E209A
Y213A
15' 30' 60'
60' no IstA
60' no IstB
60' no donor
M (kb)
10
7.5
6
5
4
3
2.5
2
1.4
1
rlx
lin
sc
(1-287)
lines not related to this study
Y170A
K126A
N188A
Y224A
Y343A R345A

Replicates

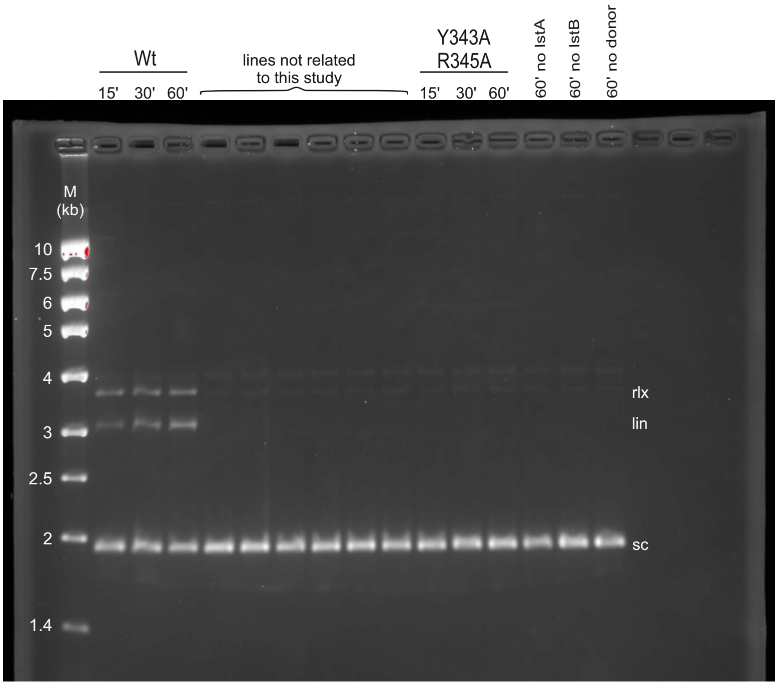

Supplementary Fig.1| Gel source data.
Un-cropped images of integration activity native agarose gels. Dotted line indicates how the gels were cropped for the final figures.

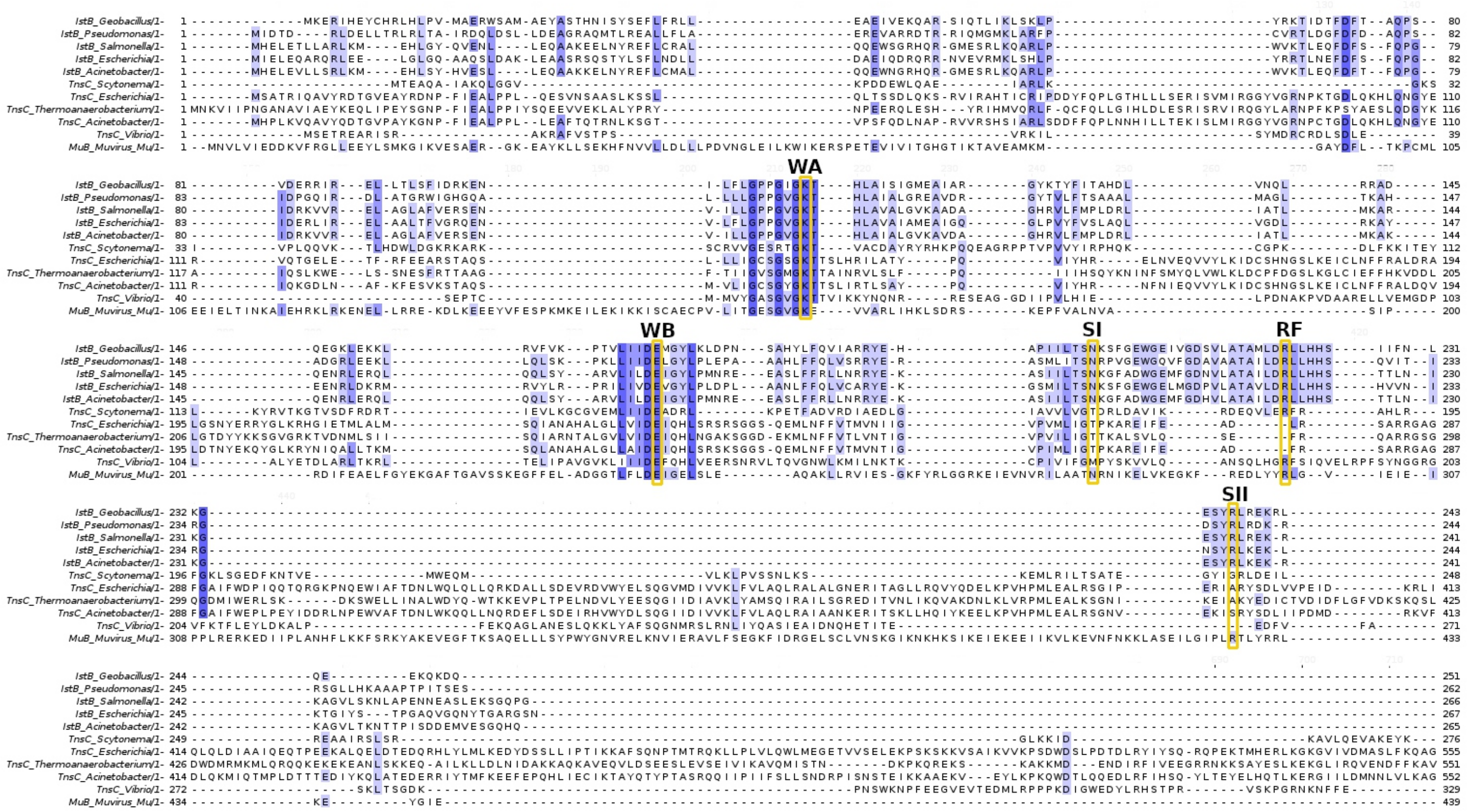

Supplementary Fig. 2| Sequence alignment of AAA+ ATPase regulators IstB, TnsC and MuB. Multiple sequence aligment of IstB, TnsC and MuB from different organisms. Critical residues for AAA+ activity (WA, Walker A; WB, Walker B; SI, Sensor I; RF, Arg Finger; and SII, Sensor II) are highlight with gold boxes using *Geobacillus stearothermophilus* IstB sequence as reference.

Supplementary Table 1| Annotated sequencing data of ten IS*5376* in vitro integration reactions. Detailed view of the sequencing results from ten integration assays shows that IS*5376* generates 5 bp direct repeats and that, at least in vitro, IstA does not appear to have a strong preference for either the left or right transposon ends. More details can be found in Spínola-Amilibia M, *et al*. Nat Commun. 2023. doi: 10.1038/s41467-023-38071-x.

Target plasmid (pSG483): black. Direct repeat: **green**. Terminal inverted repeats: orange. KanR gene: blue. Primers used for DNA sequencing: Fwd: GCAGTTTCATTTGATGCTC GATGAG, Rev: CACCTGATTGCCCGACATTATC.

Colony-Primer	DNA sequence
Col-01-Rev	NNNGGNNNGAATGNNNNNTNAGCNTANNNTTTNNNAANNNGCGNNAAATTTNNTAANTCA<br>GNTCATTTTTTNNNNNNNNNNAANTCGNNAAANNNNNTATNAATCAAAGAATAGACCGAG<br>ATAGGGTTGAGTGTNNNCCAGTTGGAACAAGAGNCCACTATTAAAGAACGTGGANTCCNA<br>CGTCAAAGGGCGAAAAACCGTCTATCAGGGCGATGGCCCANTACGTGAACCATCACCCNA<br>ATCAAGTTTTTTGGGGTCGAGGTGCCGTAAAGCACTAAATCGGAACCNTAAAGGGAGCCC<br>CCGATTTAGAGCTTGACGGGGAAAGCCGGCGAACGTGGCGAGAAAGGAAGGGAAGAAAGC<br>GAAAGGAGCGGGCGCTAGGGCGCTGGCAAGTGTAGCGGTCACGCTGCGCGTAACCACCAC<br>ACCCGCCGCGCTTAATGCGCCGCTACAGGGCGCGTCAGGTGGCACTTTTCGGGGAAATGT<br>GCGCGGAACCCCTATTTGTTTATTTTTCTAAATACATTCAAATATGTATCCGCTCATGAG<br>NCAATAACCCTGATAAATGCTTCAATAATATTGAAAAAGGAAGAGTATGAGTATTCAACA<br>TTTCCGTGTCGCCCTTATTCCCTTTTTTGCGGCATTTTGCCTTCCTGTTTTTGCTCACCC<br>AGAAACGCTGGTGAAAGTAAAAGATGCTGAAGATCAGTTGGGTGCACGAGTGGGTTACAT<br>CGAACTGGATCTCAACAGCGGTAAGATCCTTGAGAGTTTTCGCCCCGAAGAACGTTTTCC<br>AATGATGAGCACTTTTAAAGTTCTGCTATGTGGCGCGGTATTATCCCGTATTGACGCCGG<br>GCAAGAGCAACTCGGTCGCCGCATACACTATTCTCAGAATGACTTGGTTGAGTACTCACC<br>AGTCACAGAAAAGCATCTTACGGATG**GCATG**TGTTAAAGCCGATGATAAAATCCCCAATA<br>TAGCCGGAATAAAATTCCCCACTTACTCGAGCACGTTAAGGGATTTTGGTCATGAACAAT<br>AAAACTGTCTGCTTACATAAACAGTAATACAAGGGGTGTTATGAGCCATATTCAACGGGA<br>AACGTCTTGCTCTAGGCCGCGATTAAATCCAACATGGATGCTGANTATANNNNNNNNNNN<br>NN
Col-01-Fwd	NNNNNNNNTNNTGAGCGGANACATATTTGAATGTATTTAGAAAAATAAACAAATAGGGGT<br>TCCGCGCACATTTCCCCGAAAAGTGCCACCTGAAATTGTAAACGTTACTAGTTTGTTAAA<br>ATTCGCGTTAAATTTTTGTTAAATCAGCTCATTTTTTAACCAATAGGCCGAAATCGGCAA<br>AATCCCTTATAAATCAAAAGAATAGACCGAGATAGGGTTGAGTGCTCGAGTAAGTGGGGA<br>ATTTTATTCCGGCTATATTGGGGATTTTATCATCGGCTTTAACA**GCATG**ACAGTAAGAGA<br>ATTATGCAGTGCTGCCATAACCATGAGTGATAACACTGCGGCCAACTTACTTCTGACAAC<br>GATCGGAGGACCGAAGGAGCTAACCGCTTTTTTGCACAACATGGGGGATCATGTAACTCG<br>CCTTGATCGTTGGGAACCGGAGCTGAATGAAGCCATACCAAACGACGAGCGTGACACCAC<br>GATGCCTGTAGCAATGGCAACAACGTTGCGCAAACTATTAACTGGCGAACTACTTACTCT<br>AGCTTCCCGGCAACAATTAATAGACTGGATGGAGGCGGATAAAGTTGCAGGACCACTTCT<br>GCGCTCGGCCCTTCCGGCTGGCTGGTTTATTGCTGATAAATCTGGAGCCGGTGAGCGTGG<br>GTCTCGCGGTATCATTGCAGCACTGGGGCCAGATGGTAAGCCCTCCCGTATCGTAGTTAT<br>CTACACGACGGGGAGTCAGGCAACTATGGATGAACGAAATAGACAGATCGCTGAGATAGG<br>TGCCTCACTGATTAAGCATTGGTAACTGTCAGACCAAGTTTACTCATATATACTTTAGAT<br>TGATTTAAAACTTCATTTTTAATTTAAAAGGATCTAGGTGAAGATCCTTTTTGATAATCT<br>CATGACCAAAATCCCTTAACGTGAGTTTTCGTTCCACTGAGCGTCAGACCCCGTAGAAAA<br>GATCAAAGGATCTTCTTGAGATCCTTTTTTTCTGCGCGTAATCTGCTGCTTGCAAACAAA<br>AAAACCACCGCTACCAGCNGGNGGNTTGNTTGCCGGATCANAGCTACCANTCTTTTTNCG<br>NNNTAACNGGNTTCANNAGANCGCANAANACCAANACTNNNNCNANNNNANCNGNNNNNN<br>GNCCNNCACTTTNANNNANNNNNGNNN
Col-02-Rev	NNCNGAANGGNNGAATGNAAANNTNAGNNNNNNTTTNTAANTNGCNTAAATTTTNTAAAT<br>CAGCTCATTTTTTNNCCATAGGCCNAATCGNCAAAANNNTTATNAATCAAAAGAATAGAC<br>CGAGATAGGGTTGAGTGTGNNCCAGTTTGGAACAAGAGNCCACTATTAAAGAACGTGGAN

	TCCAACGTCAAAGGGCGAAAAACCGTCTATCAGGGCGATGGCCCACTACGTGAACCATCA CCCTAATCAAGTTTTTTGGGGTCGAGGTGCCGTAAAGCACTAAATCGGAACCCTAAAGGG AGCCCCCGATTTAGAGCTTGACGGGGAAAGCCGGCGAACGTGGCGAGAAAGGANNNAAGA AAGCGAAAGGAGCGGGCGCTAGGGCGCTGGCAAGTGTAGCGGTCACGCTGCGCGTAACCA CCACACCCGCCGCGCTTAATGCGCCGCTACAGGGCGCGTCAGGTGGCACTTTTCGGGGAA ATGTGCGCGGAACCCCTATTTGTTTATTTTTCTAAATACATTCAAATATGTATCCGCTCA TGAGNCAATAACCCTGATAAATGCTTCAATAATATTGAAAAAGGAAGAGTATGAGTATTC AACATTTCCGTGTCGCCCTTATTCCCTTTTTTGCGGCATTTTGCCTTCCTGTTTTTGCTC ACCCAGAAACGCTGGTGAAAGTAAAAGATGCTGAAGATCAGTTGGGTGCACGAGTGGGTT ACATCGAACTGGATCTCAACAGCGGTAAGATCCTTGAGAGTTTTCGCCCCGAAGAACGTT TTCCAATGATGAGCACTTTTAAAGTTCTGCTATGTGGCGCGGTATTATCCCGTATTGACG CCGGGCAAGAGCAACTCGGTCGCCGCATACACTATTCTCAGAATGACTTGGTTGAGTACT CACCAGTCACAGAAAAGCATCTTACGGATG**GCATG**TGTTAAAGCCGATGATAAAATCCCC AATATAGCCGGAATAAAATTCCCCACTTACTCGAGCACGTTAAGGGATTTTGGTCATGAA CAATAAAACTGTCTGCTTACATAAACAGTAATACAAGGGGTGTTATGAGCCATATTCAAC GGGAAACGTCTTGCTCTAGGCCGCGATTAAATTCCAACATGGATGCTGANTTATNNNNNN NNNNNNN
Col-02-Fwd	NNNNNNNNNNNTTNNTGAGCGGANACNTATTTGATGTATTTAGAAAAATAAACAAATAGG GGTTCCGCGCACATTTCCCCGAAAAGTGCCACCTGAAATTGTAAACGTTACTAGTTTGTT AAAATTCGCGTTAAATTTTTGTTAAATCAGCTCATTTTTTAACCAATAGGCCGAAATCGG CAAAATCCCTTATAAATCAAAAGAATAGACCGAGATAGGGTTGAGTGCTCGAGTAAGTGG GGAATTTTATTCCGGCTATATTGGGGATTTTATCATCGGCTTTAACA**GCATG**ACAGTAAG AGAATTATGCAGTGCTGCCATAACCATGAGTGATAACACTGCGGCCAACTTACTTCTGAC AACGATCGGAGGACCGAAGGAGCTAACCGCTTTTTTGCACAACATGGGGGATCATGTAAC TCGCCTTGATCGTTGGGAACCGGAGCTGAATGAAGCCATACCAAACGACGAGCGTGACAC CACGATGCCTGTAGCAATGGCAACAACGTTGCGCAAACTATTAACTGGCGAACTACTTAC TCTAGCTTCCCGGCAACAATTAATAGACTGGATGGAGGCGGATAAAGTTGCAGGACCACT TCTGCGCTCGGCCCTTCCGGCTGGCTGGTTTATTGCTGATAAATCTGGAGCCGGTGAGCG TGGGTCTCGCGGTATCATTGCAGCACTGGGGCCAGATGGTAAGCCCTCCCGTATCGTAGT TATCTACACGACGGGGAGTCAGGCAACTATGGATGAACGAAATAGACAGATCGCTGAGAT AGGTGCCTCACTGATTAAGCATTGGTAACTGTCAGACCAAGTTTACTCATATATACTTTA GATTGATTTAAAACTTCATTTTTAATTTAAAAGGATCTAGGTGAAGATCCTTTTTGATAA TCTCATGACCAAAATCCCTTAACGTGAGTTTTCGTTCCACTGAGCGTCAGACCCCGTAGA AAAGATCAAAGGATCTTCTTGAGATCCTTTTTTTCTGCGCGTAATCTGCTGCTTGCAAAC AAAAAAACCACCGCTACCAGCGGNGGTTTGTTTGNCNGGATCANANCTACCANTCTTTTT CNNNNTANTGGCTTCAGCANANCGCANATACCANNACNGNNCTTCNANNGNANCNGNANN TNNNCNN
Col-03-Rev	NCNANTNNNNCTNCNNTAAAGGGAACNAAAGCNGAGNNCNNCGNGGNNGCGNCGNTCTNN ANTAGTGNNCCGNTGAGACGNTNGNGGGGGGNCCCGGTNNCNATTCNNNNATAGTGAGTN GTATTACGCGCGCTCNCTGGCCGTCGTTTTACAACGTCGTGACTGGGAAAACCCTGGCGT TACCCAACTTAATCGCCTTGCAGCACANCCCCCTTTCGCCAGCTGGCGTAATAGCGAAGA GGCCCGCACCGATCGCCNNTTCCCAACAGTTGCGCAGNNGAATGGCGAATGGAAATTGTA AGCGTTAATATTTTGTTAAAATTCGCGTTAAATTTTTGTTAAATCAGCTCATTTTTTTAA CCAATAGGCCGAAATCGGCAAAATCCCTTATAAATCAAAAGAATAGACCGAGATAGGGTT GAGTGTTGTTCCAGTTTGGAACAAGAGTCCACTATTAAAGAACGTGGACTCCAACGTCAA AGGGCGAAAAACCGTCTATCAGGGCGATGGCCCACTACGTGAACCATCACCCTAATCAAG TTTTTTGGGGTCGAGGTGCCGTAAAGCACTAAATCGGAACCCTAAAGGGAGCCCCCGATT TAGAGCTTGACGGGGAAAGCCGGCGAACGTGGCGAGAAAGGAAGGGAAGAAAGCGAAAGG AGCGGGCGCTAGGGCGCTGGCAAGTGTAGCGGTCACGCTGCGCGTAACCACCACACCCGC CGCGCTTAATGCGCCGCTACAGGGCGCGTCAGGTGGCACTTTTCGGGGAAATGTGCGCGG AACCCCTATTTGTTTATTTTTCTAAATACATTCAAATATGTATCCGCTCATGAGACAATA ACCCTGATAAATGCTTCAATAATATTGAAAAAGGAAGAGTATGAGTATTCAACATTTCCG TGTCGCCCTTATTCCCTTTTTTGCGG**GCATT**TGTTAAAGCCGATGATAAAATCCCCAATAT AGCCGGAATAAAATTCCCCACTTACTCGAGCACGTTAAGGGATTTTGGTCATGAACAATA AAACTGTCTGCTTACATAAACAGTAATACAAGGGGTGTTATGAGCCATATTCAACGGGAA ACGTCTTGCTCTAGGCCGCGATTAAATTCCAACATGGATGCTGANNNTNNNNNNNNNNNN N
Col-03-Fwd	NNNNNNNNNNNTTNNTGAGCGGANACATATTTGAATGTATTTAGAAAAATAAACAAATAGG GGTTCCGCGCACATTTCCCCGAAAAGTGCCACCTGAAATTGTAAACGTTACTAGTTTGTT AAAATTCGCGTTAAATTTTTGTTAAATCAGCTCATTTTTTAACCAATAGGCCGAAATCGG CAAAATCCCTTATAAATCAAAAGAATAGACCGAGATAGGGTTGAGTGCTCGAGTAAGTGG

	GGAATTTTATTCCGGCTATATTGGGGATTTTATCATCGGCTTTAACA**GCATT**TTGCCTTC<br>CTGTTTTTGCTCACCCAGAAACGCTGGTGAAAGTAAAAGATGCTGAAGATCAGTTGGGTG<br>CACGAGTGGGTTACATCGAACTGGATCTCAACAGCGGTAAGATCCTTGAGAGTTTTCGCC<br>CCGAAGAACGTTTTCCAATGATGAGCACTTTTAAAGTTCTGCTATGTGGCGCGGTATTAT<br>CCCGTATTGACGCCGGGCAAGAGCAACTCGGTCGCCGCATACACTATTCTCAGAATGACT<br>TGGTTGAGTACTCACCAGTCACAGAAAAGCATCTTACGGATGGCATGACAGTAAGAGAAT<br>TATGCAGTGCTGCCATAACCATGAGTGATAACACTGCGGCCAACTTACTTCTGACAACGA<br>TCGGAGGACCGAAGGAGCTAACCGCTTTTTTGCACAACATGGGGGATCATGTAACTCGCC<br>TTGATCGTTGGGAACCGGAGCTGAATGAAGCCATACCAAACGACGAGCGTGACACCACGA<br>TGCCTGTAGCAATGGCAACAACGTTGCGCAAACTATTAACTGGCGAACTACTTACTCTAG<br>CTTCCCGGCAACAATTAATAGACTGGATGGAGGCGGATAAAGTTGCANGACCACTTCTGC<br>GCTCGGCCCTTCCGGCTGGCTGGTTTATTGCTGATAATCTGGAGCCGGTGANCGTGGGTC<br>TCGCGGTATCATTGCAGCACTGGGGCCAGATGGTAAGCCCTCCCGTATCGTANTNATCTA<br>CACGACGGGGANTCNNNAACTATGGATGAACGAAANNANANNNATCGCTGANNATNNNGC<br>CTCACTGANNNANCATTGGNNACTGNCNNANCNAGTTTACCNNCNNNNNNNACNTTTNNN<br>NTNNANTTNAAANN
Col-04-Rev	NNAAACGNGAGTTTTNGTTCCNNTGAGCNTCAGACCCCGTNGAAAANGATNAAAGNANNN<br>NTGAGANNNTTTTTCTGCGCGTAATCTGCTGCTTGCAAACAAAAAANCCNCCGNTNCCAG<br>CGGTGGTTTGTTTGCCGGATCAAGAGCTACCAACTCTTTTTCCGAAGGTAACTGGCTTCA<br>GCAGAGCGCAGATACCAAATACTGTTCTTCTAGTGTAGCCGTAGTTAGGCCACCACTTCA<br>AGAACTCTGTAGCACCGCNTACATACCTCGCTCTGCTAATCCTGTTACCAGTGGCTGCTG<br>CCAGTGGCGATAAGTCGTGTCTTACCGGGTTGGACTCAAGACGATAGTTACCGGATAAGG<br>CGCAGCGGTCGGGCTGAACGGGGGGTTCGTGCACACAGCCCAGCTTGGAGCGAACGACCT<br>ACACCGAACTGAGATACCTACAGCGTGAGCTATGAGAAAGCGCCACGCTTCCCGAAGGGA<br>GAAAGGCGGACAGGTATCCGGTAAGCGGCAGGGTCGGAACAGGAGAGCGCACGAGGGAGC<br>TTCCAGGGGGAAACGCCTGGTATCTTTATAGTCCTGTCGGGTTTCGCCACCTCTGACTTG<br>AGCGTCGATTTTTGTGATGCTCGTCAGGGGGGCGGAGCCTATGGAAAAACGCCAGCAACG<br>CGGCCTTTTTACGGTTCCTGGCCTTTTGCTGGCCTTTTGCTCACATGTTCTTTCCTGCGT<br>TATCCCCTGATTCTGTGGATAACCGTATTACCGCCTTTGAGTGAGCTGATACCGCTCGCC<br>GCAGCCGAACGACCGAGCGCAGCGAGTCAGTGAGCGAGGAAGCGGAAGAGCGCCCAATAC<br>GCAAACCGCCTCTCCCCGCGCGTTGGCCGATTCATTAATGCAGCTGGCACGACAGGTTTC<br>CCGACTGGAAAGCG**GGCAG**TGTCAAGGCCGATTATTTTTTCCCCAAAATCGCCGGTTTAA<br>AATTCCCCAGAAGGTCGAGCACGTTAAGGGATTTTGGTCATGAACAATAAAACTGTCTGC<br>TTACATAAACAGTAATACAAGGGGTGTTATGAGCCATATTCAACGGGAAACGTCTTGCTC<br>TAGGCCGCGATTAAATTCCAACATGGATGCTGANTATNNNNNNNNNNNN
Col-04-Fwd	NNNNNNNNNNNNNTGAGCGGANACNTATTTGAATGTATTTAGAAAAATAAACAAATAGGG<br>GTTCCGCGCACATTTCCCCGAAAAGTGCCACCTGAAATTGTAAACGTTACTAGTTTGTTA<br>AAATTCGCGTTAAATTTTTGTTAAATCAGCTCATTTTTTAACCAATAGGCCGAAATCGGC<br>AAAATCCCTTATAAATCAAAAGAATAGACCGAGATAGGGTTGAGTGCTCGAGTAAGTGGG<br>GAATTTTATTCCGGCTATATTGGGGATTTTATCATCGGCTTTAACA**GGCAG**TGAGCGCAA<br>CGCAATTAATGTGAGTTAGCTCACTCATTAGGCACCCCAGGCTTTACACTTTATGCTTCC<br>GGCTCGTATGTTGTGTGGAATTGTGAGCGGATAACAATTTCACACAGGAAACAGCTATGA<br>CCATGATTACGCCAAGCGCGCAATTAACCCTCACTAAAGGGAACAAAAGCTGGAGCTCCA<br>CCGCGGTGGCGGCCGCTCTAGAACTAGTGGATCCCGCTGAGGACGCTCGAGGGGGGGCCC<br>GGTACCCAATTCGCCCTATAGTGAGTCGTATTACGCGCGCTCACTGGCCGTCGTTTTACA<br>ACGTCGTGACTGGGAAAACCCTGGCGTTACCCAACTTAATCGCCTTGCAGCACATCCCCC<br>TTTCGCCAGCTGGCGTAATAGCGAAGAGGCCCGCACCGATCGCCCTTCCCAACAGTTGCG<br>CAGCCTGAATGGCGAATGGAAATTGTAAGCGTTAATATTTTGTTAAAATTCGCGTTAAAT<br>TTTTGTTAAATCAGCTCATTTTTTAACCAATAGGCCGAAATCGGCAAAATCCCTTATAA<br>ATCAAAAGAATAGACCGAGATAGGGTTGAGTGTTGTTCCAGTTTGGAACAAGAGTCCACT<br>ATTAAAGAACGTGGACTCCAACGTCAAAGGGCGAAAAACCGTCTATCNGGCGATGGCCCA<br>CTACGTGAACCATCACCCTAATCAAGTTTTTTGGGGTCGANGTGCCGTANGCACTAAATC<br>GNNNCCTAAAGGGAGCCCCCGATTTANAGCTTGACGGGGAAAGCCGGCGAACGTNNGANA<br>AAGNAAGGGAANAAAGCN
Col-05-Rev	NNNNGNAAAAAANNATNNNAAGGNANNTNNTGAGATNCTTTTTTTTTNTGNGCGNATNTGC<br>TGCTGCNAACAAAAAAAANNNNNGNTNCCANNNNGNTTNGTTTGCNGGATCAAGAGCTNCC<br>AACTCTTTTTNCGAAAGGTAACTGGCTTCAGCAGAGCGCAGATACCAAATACTGTTNTTC<br>TAGTGTAGNNGTAGTTAGGNCCNCCACTTCAAGAACTCTGTAGCACCGCNTACATACCTC<br>GCTCTGCTAATCCTGTTACCAGTGGCTGCTGCCAGTGGCGATAAGTCGTGTCTTACCGGG<br>TTGGACTCAAGACGATAGTTACCGGATAAGGCGCAGCGGTCGGGCTGAACGGGGGGTTCG<br>TGCACACAGCCCAGCTTGGAGCGAACGACNTACACCGAACTGAGATACNTACAGCGTGAG

	CTATGAGAAAGCGCCACGCTTCCCGAAGGGAGAAAGGCGGACAGGTATCCGGTAAGCGGC<br>AGGGTCGGAACAGGAGAGCGCACGAGGGAGCTTCCAGGGGGAAACGCCTGGTATCTTTAT<br>AGTCCTGTCGGGTTTCGCCACCTCTGACTTGAGCGTCGATTTTTGTGATGCTCGTCAGGG<br>GGGCGGAGCCTATGGAAAAACGCCAGCAACGCGGCCTTTTTACGGTTCCTGGCCTTTTGC<br>TGGCCTTTTGCTCACATGTTCTTTCCTGCGTTATCCCCTGATTCTGTGGATAACCGTATT<br>ACCGCCTTTGAGTGAGCTGATACCGCTCGCCGCAGCCGAACGACCGAGCGCAGCGAGTCA<br>GTGAGCGAGGAAGCGGAAGAGCGCCCAATACGCAAACCGCCTCTCCCCGCGCGTTGGCCG<br>ATTCATTAATGCAGCTGGCACGACAGGTTTCCCGACTGGAAAGCGGGCAGTGAGCGCAAC<br>GCAATTAATGTGAGTTAGCTCACTCATTAGGCACCCCAGGCTTTACACTTTATGCTTCCG<br>GCTCG**TATGT**TGTTAAAGCCGATGATAAAATCCCCAATATAGCCGGAATAAAATTCCCCA<br>CTTACTCGAGCACGTTAAGGGATTTTGGTCATGAACAATAAAACTGTCTGCTTACATAAA<br>CAGTAATACAAGGGGTGTTATGAGCCATATTCAACGGGAAACGTCTTGCTCTAGGCCGCG<br>ATTAAATTCCAACATGGATGCTGANTANNNNNNNNNNNN
Col-05-Fwd	NNNNNNNNNTTNNTGAGCGGANACATATTTGAATGTATTTAGAAAAATAAACAAATAGGG<br>GTTCCGCGCACATTTCCCCGAAAAGTGCCACCTGAAATTGTAAACGTTACTAGTTTGTTA<br>AAATTCGCGTTAAATTTTTGTTAAATCAGCTCATTTTTTAACCAATAGGCCGAAATCGGC<br>AAAATCCCTTATAAATCAAAAGAATAGACCGAGATAGGGTTGAGTGCTCGAGTAAGTGGG<br>GAATTTTATTCCGGCTATATTGGGGATTTTATCATCGGCTTTAACA**TATGT**TGTGTGGAA<br>TTGTGAGCGGATAACAATTTCACACAGGAAACAGCTATGACCATGATTACGCCAAGCGCG<br>CAATTAACCCTCACTAAAGGGAACAAAAGCTGGAGCTCCACCGCGGTGGCGGCCGCTCTA<br>GAACTAGTGGATCCCGCTGAGGACGCTCGAGGGGGGGCCCGGTACCCAATTCGCCCTATA<br>GTGAGTCGTATTACGCGCGCTCACTGGCCGTCGTTTTACAACGTCGTGACTGGGAAAACC<br>CTGGCGTTACCCAACTTAATCGCCTTGCAGCACATCCCCCTTTCGCCAGCTGGCGTAATA<br>GCGAAGAGGCCCGCACCGATCGCCCTTCCCAACAGTTGCGCAGCCTGAATGGCGAATGGA<br>AATTGTAAGCGTTAATATTTTGTTAAAATTCGCGTTAAATTTTTGTTAAATCAGCTCATT<br>TTTTTAACCAATAGGCCGAAATCGGCAAAATCCCTTATAAATCAAAAGAATAGACCGAGA<br>TAGGGTTGAGTGTTGTTCCAGTTTGGAACAAGAGTCCACTATTAAAGAACGTGGACTCCA<br>ACGTCAAAGGGCGAAAAACCGTCTATCNNGGCGATGGCCCACTACGTGAACCATCACCCT<br>AATCAAGTTTTTTGGGGTCGAGGTGCCGTANGCACTAAATCGGAACCCTAAAGGGAGCCC<br>CCGATTTAGAGCTTGACGGGGAAAGCCGGCGAACGTGGNGAGAAAGGAANGGAAGAAAGC<br>GAAAGGAGCGGGCGCTAGGGCGCTGGCAAGTGTANCGGTCACGCTGCNNGTAACCACCAC<br>ANCCGNCGNGCTTANGNNCNCTACAGGGCGCGNCNNGGTGGNNNTTTTCGGGGAAANGGN<br>GNNNNGAACCCCNATTTGNTNANTTTTNNNAAANNCNTTCNAANNN
Col-06-Rev	TTCCNNNNNGTNGNGCNGCNGAANGNNANGNAATNNNNCNNNNNTTNNNAANTNGCNTAA<br>NTTTTNTAATCAGCTCATTTTTTTNNNANNNNCGAAATCGGCAAANNCCTTATAAATCAA<br>ANANAGACCGAGATAGGGTTGAGTGTTGTNCCAGTTTGGAACAAGAGNCCACTATTAAAG<br>AACGTGGACTCCAACGTCAAAGGGCGAAAAACCGTCTATCAGGGCGATGGCCCACTACGT<br>GAACCATCACCCTAATCAAGTTTTTTGGGGTCGAGGTGCCGTAAAGCACTAAATCGGAAC<br>CNTAAAGGGAGCCCCCGATTTAGAGCTTGACGGGGAAAGCCGGCGAACGTGGCGAGAAAG<br>GAAGGGAAGAAAGCGAAAGGAGCGGGCGCTAGGGCGCTGGCAAGTGTAGCGGTCACGCTG<br>CGCGTAACCACCACACCCGCCGCGCTTAATGCGCCGCTACAGGGCGCGTCAGGTGGCACT<br>TTTCGGGGAAATGTGCGCGGAACCCCTATTTGTTTATTTTTCTAAATACATTCAAATATG<br>TATCCGCTCATGAGACAATAACCCTGATAAATGCTTCAATAATATTGAAAAAGGAAGAGT<br>ATGAGTATTCAACATTTCCGTGTCGCCCTTATTCCCTTTTTTGCGGCATTTTGCCTTCCT<br>GTTTTTGCTCACCCAGAAACGCTGGTGAAAGTAAAAGATGCTGAAGATCAGTTGGGTGCA<br>CGAGTGGGTTACATCGAACTGGATCTCAACAGCGGTAAGATCCTTGAGAGTTTTCGCCCC<br>GAAGAACGTTTTCCAATGATGAGCACTTTTAAAGTTCTGCTATGTGGCGCGGTATTATCC<br>CGTATTGACGCCGGGCAAGAGCAACTCGGTCGCCGCATACACTATTCTCAGAATGACTTG<br>GTTGAGTACTCACCAGTCACAGAAAAGCATCTTACGGATG**GCATG**TGTCAAGGCCGATTA<br>TTTTTTCCCCAAAATCGCCGGTTTAAAATTCCCCAGAAGGTCGAGCACGTTAAGGGATTT<br>TGGTCATGAACAATAAAACTGTCTGCTTACATAAACAGTAATACAAGGGGTGTTATGAGC<br>CATATTCAACGGGAAACGTCTTGCTCTAGGCCGCGATTAAATTCCAACATGGATGCTGAN<br>TATNNNNNNNNNNNN
Col-06-Fwd	NNNNNNNNNNNNNNGAGCGGANACNTATTTGAATGTATTTAGAAAAATAAACAAATAGGG<br>GTTCCGCGCACATTTCCCCGAAAAGTGCCACCTGAAATTGTAAACGTTACTAGTTTGTTA<br>AAATTCGCGTTAAATTTTTGTTAAATCAGCTCATTTTTTAACCAATAGGCCGAAATCGGC<br>AAAATCCCTTATAAATCAAAAGAATAGACCGAGATAGGGTTGAGTGCTCGACCTTCTGGG<br>GAATTTTAAACCGGCGATTTTGGGGAAAAAATAATCGGCCTTGACA**GCATG**ACAGTAAGA<br>GAATTATGCAGTGCTGCCATAACCATGAGTGATAACACTGCGGCCAACTTACTTCTGACA<br>ACGATCGGAGGACCGAAGGAGCTAACCGCTTTTTTGCACAACATGGGGGATCATGTAACT<br>CGCCTTGATCGTTGGGAACCGGAGCTGAATGAAGCCATACCAAACGACGAGCGTGACACC

	ACGATGCCTGTAGCAATGGCAACAACGTTGCGCAAACTATTAACTGGCGAACTACTTACT<br>CTAGCTTCCCGGCAACAATTAATAGACTGGATGGAGGCGGATAAAGTTGCAGGACCACTT<br>CTGCGCTCGGCCCTTCCGGCTGGCTGGTTTATTGCTGATAAATCTGGAGCCGGTGAGCGT<br>GGGTCTCGCGGTATCATTGCAGCACTGGGGCCAGATGGTAAGCCCTCCCGTATCGTAGTT<br>ATCTACACGACGGGGAGTCAGGCAACTATGGATGAACGAAATAGACAGATCGCTGAGATA<br>GGTGCCTCACTGATTAAGCATTGGTAACTGTCAGACCAAGTTTACTCATATATACTTTAG<br>ATTGATTTAAAACTTCATTTTTAATTTAAAAGGATCTAGGTGAAGATCCTTTTTGATAAT<br>CTCATGACCAAAATCCCTTAACGTGAGTTTTCGTTCCACTGAGCGTCAGACCCCGTAGAA<br>AAGATCAAAGGATCTTCTTGAGATCCTTTTTTTCTGCGCGTAATCTGCTGCTTGCAAACA<br>AAAAAACCACCGCTACCAGCGGTGGNTTGTTTGCCGGATCANAGCTACCANTCTTTNNNN<br>NNTANTGGNTTCAGCAGAGCGCAGATNCNANTACTNNNNNNANNGNANNNTANNNNGCCA<br>NCNANTTCANAACNNNNNANCNCN
Col-07-Rev	NNGCAGNNNNNNCCCTTTNNCAGCTGGCGTAATAGCGAAGAGNCCGCNNCGATCNCNNNN<br>NNNAGTGNGCAGCCTGAATGGCGAATGGAAATGTAAGNNTAATATTTGTTAAAATTCGCG<br>TTAAATTTTTGTTAAATCAGCTCATTTTTTTNANCCAATAGGCCGAAATCGGCAAAATNC<br>CNTTATAAATCAAAAGAATAGACCGAGATAGGGTTGAGTGTTGTTCCAGTTTGGAACAAG<br>AGTCCACTATTAAAGAACGTGGACTCCAACGTCAAAGGGCGAAAAACCGTCTATCAGGGC<br>GATGGCCCACTACGTGAACCATCACCCTAATCAAGTTTTTTGGGGTCGAGGTGCCGTAAA<br>GCACTAAATCGGAACCCTAAAGGGAGCCCCCGATTTAGAGCTTGACGGGGAAAGCCGGCG<br>AACGTGGCGAGAAAGGAAGGGAAGAAAGCGAAAGGAGCGGGCGCTAGGGCGCTGGCAAGT<br>GTAGCGGTCACGCTGCGCGTAACCACCACACCCGCCGCGCTTAATGCGCCGCTACAGGGC<br>GCGTCAGGTGGCACTTTTCGGGGAAATGTGCGCGGAACCCCTATTTGTTTATTTTTCTAA<br>ATACATTCAAATATGTATCCGCTCATGAGACAATAACCCTGATAAATGCTTCAATAATAT<br>TGAAAAAGGAAGAGTATGAGTATTCAACATTTCCGTGTCGCCCTTATTCCCTTTTTTGCG<br>GCATTTTGCCTTCCTGTTTTTGCTCACCCAGAAACGCTGGTGAAAGTAAAAGATGCTGAA<br>GATCAGTTGGGTGCACGAGTGGGTTACATCGAACTGGATCTCAACAGCGGTAAGATCCTT<br>GAGAGTTTTCGCCCCGAAGAACGTTTTCCAATGATGAGCACTTTTAAAGTTCTGCTATGT<br>GGCGCGGTATTATCCCGTATTG**ACGCC**TGTCAAGGCCGATTATTTTTTCCCCAAAATCGC<br>CGGTTTAAAATTCCCCAGAAGGTCGAGCACGTTAAGGGATTTTGGTCATGAACAATAAAA<br>CTGTCTGCTTACATAAACAGTAATACAAGGGGTGTTATGAGCCATATTCAACGGGAAACG<br>TCTTGCTCTAGGCCGCGATTAAATCCAACATGGATGCTGNTNTANNNNNNNNNNCNNNN
Col-07-Fwd	NNNNNNNNNNNTCNTGAGCGGATACNTATTTGAATGTATTTAGAAAAATAAACAAATAGG<br>GGTTCCGCGCACATTTCCCCGAAAAGTGCCACCTGAAATTGTAAACGTTACTAGTTTGTT<br>AAAATTCGCGTTAAATTTTTGTTAAATCAGCTCATTTTTTAACCAATAGGCCGAAATCGG<br>CAAAATCCCTTATAAATCAAAAGAATAGACCGAGATAGGGTTGAGTGCTCGAGTAAGTGG<br>GGAATTTTATTCCGGCTATATTGGGGATTTTATCATCGGCTTTAACA**ACGCC**GGGCAAGA<br>GCAACTCGGTCGCCGCATACACTATTCTCAGAATGACTTGGTTGAGTACTCACCAGTCAC<br>AGAAAAGCATCTTACGGATGGCATGACAGTAAGAGAATTATGCAGTGCTGCCATAACCAT<br>GAGTGATAACACTGCGGCCAACTTACTTCTGACAACGATCGGAGGACCGAAGGAGCTAAC<br>CGCTTTTTTGCACAACATGGGGGATCATGTAACTCGCCTTGATCGTTGGGAACCGGAGCT<br>GAATGAAGCCATACCAAACGACGAGCGTGACACCACGATGCCTGTAGCAATGGCAACAAC<br>GTTGCGCAAACTATTAACTGGCGAACTACTTACTCTAGCTTCCCGGCAACAATTAATAGA<br>CTGGATGGAGGCGGATAAAGTTGCAGGACCACTTCTGCGCTCGGCCCTTCCGGCTGGCTG<br>GTTTATTGCTGATAAATCTGGAGCCGGTGAGCGTGGGTCTCGCGGTATCATTGCAGCACT<br>GGGGCCAGATGGTAAGCCCTCCCGTATCGTAGTTATCTACACGACGGGGAGTCAGGCAAC<br>TATGGATGAACGAAATAGACAGATCGCTGAGATAGGTGCCTCACTGATTAAGCATTGGTA<br>ACTGTCAGANCAAGTTTACTCATATATACTTTAGATTGATTTAAAACTTCATTTTTAATT<br>TAAAAGGATCTAGGTGAAGATCCTTTTTGATAATCTCATGACCAAAATCCCTTAACGTGA<br>GTTTTCGTTCCACTGANCGTCAGACCCCGTANAAAGATCAAAGGATCTTNNNNNATCCTT<br>TTTTCTGCNCGTAATCNGCTGCTTGCAACAAAAAACCANCGCTACCNNCGGNNGNTTNGN
Col-08-Rev	NTTTTCGGGGNAAAANGNNNCGNCGGAAACCCNTNNTTGTTTATTTTCTAAATNCNTTNA<br>ATATGTATCCGCTCANGANNNANNCCTGATAAATGNTCAATAATNTGAAAAAGGAAGAGT<br>ATGAGTATTCANCNNTTTCCGNGTCGNNTTANNCNNTTTTTGCGGCATTTTGCNNTCCTG<br>TTTTTGCTCACCCAGAAACGCTGGTGAAAGTAAAAGATGCTGAAGATCAGTTGGGTGCAC<br>GAGTGGGTTACATCGAACTGGATCTCAACAGCGGTAAGATCCTTGAGAGTTTTCGCCCCG<br>AAGAACGTTTTCCAATGATGAGCACTTTTAAAGTTCTGCTATGTGGCGCGGTATTATCCC<br>GTATTGACGCCGGGCAAGAGCAACTCGGTCGCCGCATACACTATTCTCAGAATGACTTGG<br>TTGAGTACTCACCAGTCACAGAAAAGCATCTTACGGATGGCATGACAGTAAGAGAATTAT<br>GCAGTGCTGCCATAACCATGAGTGATAACACTGCGGCCAACTTACTTCTGACAACGATCG<br>GAGGACCGAAGGAGCTAACCGCTTTTTTGCACAACATGGGGGATCATGTAACTCGCCTTG<br>ATCGTTGGGAACCGGAGCTGAATGAAGCCATACCAAACGACGAGCGTGACACCACGATGC

	CTGTAGCAATGGCAACAACGTTGCGCAAACTATTAACTGGCGAACTACTTACTCTAGCTT<br>CCCGGCAACAATTAATAGACTGGATGGAGGCGGATAAAGTTGCAGGACCACTTCTGCGCT<br>CGGCCCTTCCGGCTGGCTGGTTTATTGCTGATAAATCTGGAGCCGGTGAGCGTGGGTCTC<br>GCGGTATCATTGCAGCACTGGGGCCAGATGGTAAGCCCTCCCGTATCGTAGTTATCTACA<br>CGACGGGGAGTCAGGCAACTATGGATGAACGAAATAGACAGATCGCTGA**GATAG**TGTCAA<br>GGCCGATTATTTTTTCCCCAAAATCGCCGGTTTAAAATTCCCCAGAAGGTCGAGCACGTT<br>AAGGGATTTTGGTCATGAACAATAAAACTGTCTGCTTACATAAACAGTAATACAAGGGGT<br>GTTANNAGNCATATTCAACGGGAAACGTCTTGCTNTAGGCCGCGATTAAATTCCAACATG<br>GATGCNGNNTNNNNNNNNNNNNNNNNNNNNNC
Col-08-Fwd	NNNNNNNNNNNNNNNNNNNNCGGANACATATTTGAATGTATTTAGAAAAATAAACAAATA<br>GGGGTTCCGCGCACATTTCCCCGAAAAGTGCCACCTGAAATTGTAAACGTTACTAGTTTG<br>TTAAAATTCGCGTTAAATTTTTGTTAAATCAGCTCATTTTTTAACCAATAGGCCGAAATC<br>GGCAAAATCCCTTATAAATCAAAAGAATAGACCGAGATAGGGTTGAGTGCTCGAGTAAGT<br>GGGGAATTTTATTCCGGCTATATTGGGGATTTTATCATCGGCTTTAACA**GATAG**GTGCCT<br>CACTGATTAAGCATTGGTAACTGTCAGACCAAGTTTACTCATATATACTTTAGATTGATT<br>TAAAACTTCATTTTTAATTTAAAAGGATCTAGGTGAAGATCCTTTTTGATAATCTCATGA<br>CCAAAATCCCTTAACGTGAGTTTTCGTTCCACTGAGCGTCAGACCCCGTAGAAAAGATCA<br>AAGGATCTTCTTGAGATCCTTTTTTTCTGCGCGTAATCTGCTGCTTGCAAACAAAAAAAC<br>CACCGCTACCAGCGGTGGTTTGTTTGCCGGATCAAGAGCTACCAACTCTTTTTCCGAAGG<br>TAACTGGCTTCAGCAGAGCGCAGATACCAAATACTGTTCTTCTAGTGTAGCCGTAGTTAG<br>GCCACCACTTCAAGAACTCTGTAGCACCGCCTACATACCTCGCTCTGCTAATCCTGTTAC<br>CAGTGGCTGCTGCCAGTGGCGATAAGTCGTGTCTTACCGGGTTGGACTCAAGACGATAGT<br>TACCGGATAANGCGCAGCGGTCGGGCTGAACGGGGGGTTCGTGCACACAGCCCAGCTTNN<br>NCGAACGACCTACACCGAACTGAGATACCTACAGCGTGAGCTATGAGAAAGCGCCACGCT<br>TCCCGAAAGGGAGAAAGGCGGACAGGTATCCGGTAAGCGGCNNGGTCGGAACAGGANAGC<br>GCACGANGGANCTTCCAGGGGGAAACGCCTGGNATCTTTATAGTCCTGTCGGGTTTCGCC<br>NCCTCTGACTTGAGCGTCGATTTTNTGATGCTCGTCNNGGGCGNNCCTATGGAAAACNCN<br>GCANGCGNNTTTTACGNNCNNNNTTGCTNNNTTTGCTCNCANNTCTTNCNGNGTTATCCC<br>NGATNNNGNNGGANNANCGNATTACNNNN
Col-09-Rev	NNNNNNNNCTTTANNNNTTCCNNGNNNGTNNTGTTGNNNNGGNNNNTNNAGGCGGATANC<br>NATTTNNCNCNGNAANAGCTATGNNCATGNNTACNCCAAGCGCGCNNNACCCTCNNNAAA<br>GGGANCAAAAGCTGGAGNNCNNCCCGCGNTGGCGNCCGNTCTAGAACTAGTGGATCCCGCT<br>GAGGACGCTCGAGGGGGGGCCCGGTACCCAATTCGCCCTATAGTGAGTCGTATTACGCGC<br>GCTCACTGGCCGTCGTTTTACAACGTCGTGACTGGGAAAACCCTGGCGTTACCCAACTTA<br>ATCGCCTTGCAGCACATCCCCCTTTCGCCAGCTGGCGTAATAGCGAAGAGGCCCGCACCG<br>ATCGCCCTTCCCAACAGTTGCGCAGCNTGAATGGCGAATGGAAATTGTAAGCGTTAATAT<br>TTTGTTAAAATTCGCGTTAAATTTTTGTTAAATCAGCTCATTTTTTAACCAATAGGCCG<br>AAATCGGCAAAATCCCTTATAAATCAAAAGAATAGACCGAGATAGGGTTGAGTGTTGTTC<br>CAGTTTGGAACAAGAGTCCACTATTAAAGAACGTGGACTCCAACGTCAAAGGGCGAAAAA<br>CCGTCTATCAGGGCGATGGCCCACTACGTGAACCATCACCCTAATCAAGTTTTTTGGGGT<br>CGAGGTGCCGTAAAGCACTAAATCGGAACCCTAAAGGGAGCCCCCGATTTAGAGCTTGAC<br>GGGGAAAGCCGGCGAACGTGGCGAGAAAGGAAGGGAAGAAAGCGAAAGGAGCGGGCGCTA<br>GGGCGCTGGCAAGTGTAGCGGTCACGCTGCGCGTAACCACCACACCCGCCGCGCTTAATG<br>CGCCGCTACAGGGCGCGTCAGGTGGCACTTTTCGGGGAAATGTGCGCGGAACCCCTATTT<br>GTTTATTTTTCTAAATACATTCAAATATGTATCCGCTCATGAGACAATAACCCTGATAAA<br>TGCTTCAATAATATTGAAAAAGGAA**GAGTA**TGTCAAGGCCGATTATTTTTTCCCCAAAAT<br>CGCCGGTTTAAAATTCCCCAGAAGGTCGAGCACGTTAAGGGATTTTGGTCATGAACAATA<br>AAACTGTCTGCTTACATAAACAGTAATACAAGGGGTGTTATGAGCCATATTCAACGGGAA<br>ACGTCTTGCTCTAGGCCGCGATTAAATTCCAACATGGATGCTGNNNNNNNNNNNNNNNNN<br>NN
Col-09-Fwd	NNNNNNNNTTNNTGAGCGGANACATATTTGAATGTATTTAGAAAAATAAACAAATAGGGG<br>TTCCGCGCACATTTCCCCGAAAAGTGCCACCTGAAATTGTAAACGTTACTAGTTTGTTAA<br>AATTCGCGTTAAATTTTTGTTAAATCAGCTCATTTTTTAACCAATAGGCCGAAATCGGCA<br>AAATCCCTTATAAATCAAAAGAATAGACCGAGATAGGGTTGAGTGCTCGACCTTCTGGGG<br>AATTTTAAACCGGCGATTTTGGGGAAAAAATAATCGGCCTTGACA**GAGTA**TGAGTATTCA<br>ACATTTCCGTGTCGCCCTTATTCCCTTTTTTGCGGCATTTTGCCTTCCTGTTTTTGCTCA<br>CCCAGAAACGCTGGTGAAAGTAAAAGATGCTGAAGATCAGTTGGGTGCACGAGTGGGTTA<br>CATCGAACTGGATCTCAACAGCGGTAAGATCCTTGAGAGTTTTCGCCCCGAAGAACGTTT<br>TCCAATGATGAGCACTTTTAAAGTTCTGCTATGTGGCGCGGTATTATCCCGTATTGACGC<br>CGGGCAAGAGCAACTCGGTCGCCGCATACACTATTCTCAGAATGACTTGGTTGAGTACTC<br>ACCAGTCACAGAAAAGCATCTTACGGATGGCATGACAGTAAGAGAATTATGCAGTGCTGC

	CATAACCATGAGTGATAACACTGCGGCCAACTTACTTCTGACAACGATCGGAGGACCGAA<br>GGAGCTAACCGCTTTTTTGCACAACATGGGGGATCATGTAACTCGCCTTGATCGTTGGGA<br>ACCGGAGCTGAATGAAGCCATACCAAACGACGAGCGTGACACCACGATGCCTGTAGCAAT<br>GGCAACAACGTTGCGCAAACTATTAACTGGCGAACTACTTACTCTAGCTTCCCGGCAACA<br>ATTAATAGACTGGATGGAGGCGGATAAAGTTGCAGGACCACTTCTGCGCTCGGCCCTTCC<br>GGCTGGCTGGTTTATTGCTGATAAATCTGGAGCCCGNNGAGCGTGGGTCTCGCGGTATCA<br>TTGCAGCACTGGGGNCAGATGGTAAGCCCTCCCGTATCGTANTTATCTACNCGACGGGGN<br>NTCNNNANTATGGNATGANNAAANANACNGATCGCTGAGATNGGTNCNCNCNGATNNCAT<br>TGGNNACNGTNNNANCNAGTTNNNCNNNNNNNNNNNTTNNANNNNN
Col-10-Rev	NNNNTNNNNGGAAATNNNGANNNGNNANNNATTTCNNNNCCNGNAAACAGNTATGNCCAN<br>GNTANNCNAGCNNNNNNNCCTCNNTAANGGNNNNNGCNGNGNTCNNCCGCGTGNNNNGCT<br>CTAGNANTAGTGGATCCCGCTGAGGACGCTNGAGGGGGGCCCGGTACCCAATTCGCCCTA<br>TAGTGAGTCGTATTACGCGCGCTCACTGGCCGTCGTTTTACAACGTCGTGACTGGGAAAA<br>CCCTGGCGTTACCCAACTTAATCGCCTTGCAGCACATCCCCCTTTCGCCAGCTGGCGTAA<br>TAGCGAAGAGGCCCGCACCGATCGCCCTTCCCAACAGTTGCGCAGCNTGAATGGCGAATG<br>GAAATTGTAAGCGTTAATATTTTGTTAAAATTCGCGTTAAATTTTTGTTAAATCAGCTCA<br>TTTTTTAACCAATAGGCCGAAATCGGCAAAATCCCTTATAAATCAAAAGAATAGACCGA<br>GATAGGGTTGAGTGTTGTTCCAGTTTGGAACAAGAGTCCACTATTAAAGAACGTGGACTC<br>CAACGTCAAAGGGCGAAAAACCGTCTATCAGGGCGATGGCCCACTACGTGAACCATCACC<br>CTAATCAAGTTTTTTGGGGTCGAGGTGCCGTAAAGCACTAAATCGGAACCCTAAAGGGAG<br>CCCCCGATTTAGAGCTTGACGGGGAAAGCCGGCGAACGTGGCGAGAAAGGAAGGGAAGAA<br>AGCGAAAGGAGCGGGCGCTAGGGCGCTGGCAAGTGTAGCGGTCACGCTGCGCGTAACCAC<br>CACACCCGCCGCGCTTAATGCGCCGCTACAGGGCGCGTCAGGTGGCACTTTTCGGGGAAA<br>TGTGCGCGGAACCCCTATTTGTTTATTTTTCTAAATACATTCAAATATGTATCCGCTCAT<br>GAGACAATAACCCTGATAAATGCTTCAATAATATTGAAAAAGGAA**GAGTA**TGTCAAGGCC<br>GATTATTTTTTCCCCAAAATCGCCGGTTTAAAATTCCCCAGAAGGTCGAGCACGTTAAGG<br>GATTTTGGTCATGAACAATAAAACTGTCTGCTTACATAAACAGTAATACAAGGGGTGTTA<br>TGAGCCATATTCAACGGGAAACGTCTTGCTCTAGGCCGCGATTAAATTCCAACATGGATG<br>CTGATTATNNNNNNNNNNNNNN
Col-10-Fwd	NNNNNNNNNNTNNTGAGCGGANACNTATTTGAATGTATTTAGAAAAATAAACAAATAGGG<br>GTTCCGCGCACATTTCCCCGAAAAGTGCCACCTGAAATTGTAAACGTTACTAGTTTGTTA<br>AAATTCGCGTTAAATTTTTGTTAAATCAGCTCATTTTTTAACCAATAGGCCGAAATCGGC<br>AAAATCCCTTATAAATCAAAAGAATAGACCGAGATAGGGTTGAGTGCTCGACCTTCTGGG<br>GAATTTTAAACCGGCGATTTTGGGGAAAAAATAATCGGCCTTGACA**GAGTA**TGAGTATTC<br>AACATTTCCGTGTCGCCCTTATTCCCTTTTTTGCGGCATTTTGCCTTCCTGTTTTTGCTC<br>ACCCAGAAACGCTGGTGAAAGTAAAAGATGCTGAAGATCAGTTGGGTGCACGAGTGGGTT<br>ACATCGAACTGGATCTCAACAGCGGTAAGATCCTTGAGAGTTTTCGCCCCGAAGAACGTT<br>TTCCAATGATGAGCACTTTTAAAGTTCTGCTATGTGGCGCGGTATTATCCCGTATTGACG<br>CCGGGCAAGAGCAACTCGGTCGCCGCATACACTATTCTCAGAATGACTTGGTTGAGTACT<br>CACCAGTCACAGAAAAGCATCTTACGGATGGCATGACAGTAAGAGAATTATGCAGTGCTG<br>CCATAACCATGAGTGATAACACTGCGGCCAACTTACTTCTGACAACGATCGGAGGACCGA<br>AGGAGCTAACCGCTTTTTTGCACAACATGGGGGATCATGTAACTCGCCTTGATCGTTGGG<br>AACCGGAGCTGAATGAAGCCATACCAAACGACGAGCGTGACACCACGATGCCTGTAGCAA<br>TGGCAACAACGTTGCGCAAACTATTAACTGGCGAACTACTTACTCTAGCTTCCCGGCAAC<br>AATTAATAGACTGGATGGAGGCGGATAAAGTTGCNNACCACTTCTGCGCTCGGCCCTTCC<br>GGCTGGCTGGTTTATTGCTGATAAATCTGGAGCCGGTGANCGTGGGTCTCGCGGTATCAT<br>TGCAGCACTGGGGCCAGATGGTAAGCCCTCCCGTATCGTANTTATCTACNCGANGGGGNN<br>TCNNGNAACTATGGNTGANGAAATANNCNGNTCGCNGANNNAGNNGCNNCACNGANTA
